# Phenotypic and genotypic characterization of antimicrobial resistance in coagulase-negative staphylococci from bone lesions in broiler chickens

**DOI:** 10.1186/s12917-026-05584-8

**Published:** 2026-05-26

**Authors:** G. M. Szafraniec, D. Chrobak-Chmiel, K. Adamczyk, K. Sułecki, B. Dolka

**Affiliations:** 1 Department of Pathology and Veterinary Diagnostics, Institute of Veterinary Medicine, Warsaw University of Life Sciences – SGGW, Nowoursynowska 159c St, Warsaw, 02-776 Poland; 2 Department of Preclinical Sciences, Institute of Veterinary Medicine, Warsaw University of Life Sciences – SGGW, Nowoursynowska 159c St, Warsaw, 02-776 Poland; 3 Independent Researcher, Warsaw, Poland

**Keywords:** *Staphylococcus*, Coagulase-negative staphylococci, Antimicrobial resistance, BCO, Lameness, Femoral head necrosis, Poultry, Chicken

## Abstract

**Background:**

Lameness in broiler chickens, frequently caused by bacterial chondronecrosis with osteomyelitis (BCO), poses a major challenge to intensive poultry production due to its adverse impact on animal welfare and farm economics. While *Staphylococcus aureus* has traditionally been considered the principal staphylococcal pathogen in poultry, coagulase-negative staphylococci (CoNS) are increasingly implicated in skeletal infections. This study aimed to characterize the phenotypic and genotypic antimicrobial resistance profiles of 93 CoNS isolates representing 12 *Staphylococcus* species. Isolates were recovered from bone and joint lesions of lame broiler chickens from 25 commercial flocks in Poland and were characterized using disk diffusion testing, PCR-based detection of selected resistance genes, pulsed-field gel electrophoresis (PFGE), and exploratory whole-genome sequencing (WGS).

**Results:**

Disk diffusion testing and PCR screening revealed a high prevalence of resistance to penicillin, doxycycline, and erythromycin, with *bl*a_Z_ (37.6%; 35/93), *lnu*(A) (37.6%; 35/93), *tet*(L) (25.8%; 24/93), *tet*(M) (22.6%; 21/93), and *erm*(C) (12.9%; 12/93) being the most commonly detected determinants. The *mecA* gene, conferring resistance to β-lactams, was detected in a subset of isolates (16.1%; 15/93) although phenotypic resistance did not always correlate with *mecA* presence. Femoral-head lesions (FHN/FHT/FHS; *n* = 50) yielded isolates with resistance profiles broadly similar to those observed in the overall collection. PFGE of *S. cohnii* demonstrated genetic heterogeneity within the analyzed subset. Whole-genome analysis of *S. cohnii* strain SC27 identified multiple AMR-associated determinants and mobile genetic elements.

**Conclusions:**

These findings indicate that CoNS associated with broiler skeletal lesions can harbor diverse AMR determinants linked to several widely used antimicrobial classes. Our findings are primarily relevant from an epidemiological and surveillance perspective. The study also adds new genomic and epidemiological context to current knowledge of AMR in broiler-associated CoNS and supports their inclusion in ongoing veterinary and One Health-oriented surveillance efforts.

**Supplementary Information:**

The online version contains supplementary material available at 10.1186/s12917-026-05584-8.

## Introduction

Lameness is an important and consistently relevant issue in modern broiler production, with consequences for animal welfare, productivity, and economic performance. Affected birds experience pain, which leads to reduced mobility, poorer feed intake, and as a result, smaller weight gains, increased mortality, and overall worse economic outcomes [1–5]. Lameness in broiler chickens is a complex issue that includes genetic, environmental, nutritional, and infectious factors. In modern broilers, rapid body weight and muscle gain may occur before the skeleton has fully developed, exposing immature bones and growth plates to increased biomechanical forces and thereby predisposing birds to skeletal damage and lameness. Among infectious factors, bacterial chondronecrosis with osteomyelitis (BCO), also known as femoral head necrosis (FHN), is considered the leading cause of lameness in chickens [4]. BCO most commonly occurs in fast-growing broiler chickens and is characterized by necrotic lesions mainly at the proximal ends of femur and tibiotarsal bones, as well as thoracic vertebrae – sites associated with biomechanical stress where damage to the structure of the bone and its vasculature can lead to osteomyelitis [6].

Among staphylococci, *Staphylococcus aureus* is well known for causing local, usually chronic, and sometimes systemic infections in poultry. It has traditionally been described as the main species of bacteria involved in BCO in chickens [3, 7, 8]. However, recent research indicates the growing role of coagulase-negative staphylococci (CoNS) as important opportunistic pathogens in poultry infections [9, 10]. CoNS are ubiquitous in the hatchery and farm environments and possess the ability to form biofilm and acquire diverse antimicrobial resistance genes, complicating effective treatment and control [11–13].

Antimicrobial resistance (AMR) is a global concern, and its prevalence among bacterial pathogens in food-producing animals is particularly relevant to both veterinary and human health [14–17]. In broiler production, the extensive, routine, and often prophylactic use of antibiotics creates strong selective pressure that favors the emergence and spread of resistant bacteria, including staphylococci [18–21]. AMR in staphylococci is commonly categorized according to the extent of non-susceptibility across antimicrobial classes. Multidrug-resistant (MDR) strains are defined as non-susceptible to at least one agent in three or more antimicrobial classes, while extensively drug-resistant (XDR) strains remain susceptible to no more than two classes, and pandrug-resistant (PDR) strains are non-susceptible to all agents in all antimicrobial classes [22]. Such resistance profiles pose a significant challenge for effective disease management as treatment options become increasingly limited [19, 23].

In the context of broiler skeletal disease, the clinical relevance of AMR should be interpreted cautiously. Birds with advanced lameness or bone lesions are often culled rather than treated. Nevertheless, the detection of resistant staphylococci remains relevant from an epidemiological and surveillance perspective, as these bacteria may act as reservoirs of antimicrobial resistance determinants within poultry production systems [24, 25].

Resistance to key antibiotic classes such as β-lactams, macrolides, tetracyclines, and aminoglycosides is increasingly reported among staphylococcal isolates from poultry environments [20, 26]. Particular attention has been paid to methicillin resistance in staphylococci because it predicts resistance to most β-lactam antibiotics. Although methicillin itself is no longer used in clinical practice, terms such as methicillin-resistant *Staphylococcus aureus* (MRSA) and methicillin-resistant coagulase-negative staphylococci (MRCoNS) remain in use to denote resistance associated mainly with the *mecA* gene and the corresponding reduced susceptibility to isoxazolyl penicillins such as oxacillin or cloxacillin, as well as to most cephalosporins and other β-lactams, with a few recognized exceptions [27]. In veterinary medicine, methicillin resistance is also important in other staphylococcal species, including methicillin-resistant *Staphylococcus pseudintermedius* (MRSP), which represents a well-recognized concern, particularly in companion animals [28]. However, while methicillin resistance has been studied extensively in *S. aureus* and *S. pseudintermedius*, less attention has been paid to CoNS, particularly with regard to their species distribution, resistance phenotypes, and carriage of selected antimicrobial resistance determinants.

This study aimed to provide new insights into the prevalence and characteristics of methicillin-resistant and multidrug-resistant CoNS isolated from the skeletal lesions of lame broiler chickens. It investigates the resistance profiles of staphylococci associated with BCO and other major skeletal lesions in broilers, with particular emphasis on species distribution, phenotypic resistance patterns, and the carriage of selected antimicrobial resistance determinants.

## Materials and methods

### *Staphylococcus* strains

This study involved previously obtained *Staphylococcus* isolates recovered from tissue samples from broiler chicken carcasses collected through daily on-farm flock health monitoring [29]. In total, 93 CoNS strains were isolated from bone and joint lesions of 2-6-week-old broiler chickens originating from 25 commercial flocks, predominantly in eastern Poland. Carcasses were submitted for necropsy to the Department of Pathology and Veterinary Diagnostics, Institute of Veterinary Medicine, Warsaw University of Life Sciences – SGGW by on-farm veterinarians. All tissue samples and bacterial strains were obtained post-mortem from birds that had died on the farm due to disease and/or had been euthanized on-farm by veterinarians or trained farm personnel for welfare reasons (e.g., lameness), in accordance with standard agricultural practices and applicable national and EU legislation. The investigators were not involved in any euthanasia procedures; no birds were euthanized by the authors specifically for the purposes of this study, and all examinations were performed post-mortem. Information on prior antimicrobial treatment at flock or individual level was not consistently available and was therefore not included in the analysis.

To minimize the risk of environmental or skin-derived contamination, samples were collected aseptically directly from lesion sites in fresh carcasses. In addition to routine culture for staphylococci, lesion material was inoculated onto selective and differential media, including Enterococcosel agar and MacConkey agar (GRASO Biotech, Poland), to detect other bacterial groups potentially involved in skeletal lesions in broiler chickens. In a small proportion of cases, mixed cultures were obtained, most commonly with *Enterococcus* spp., whereas co-occurrence with *Escherichia coli* was observed less frequently. In most lesion samples yielding staphylococci, these organisms represented the predominant bacterial growth recovered from the lesions.

The identification of *Staphylococcus* isolates to the species level and classification of osteoarticular lesions have been described in detail in our previous study [29]. In short, to minimize the risk of contamination, swab samples from bone marrow and synovial fluid were collected aseptically directly from lesion sites in fresh carcasses and inoculated onto Columbia Agar with 5% sheep blood (GRASO Biotech, Poland). All bacterial cultures were screened using colony morphology, Gram staining, and basic bacteriological tests (catalase, oxidase, and coagulase). Isolates meeting the criteria for staphylococci were then subjected to DNA extraction, followed by PCR amplification and sequencing of the 16 S rRNA gene. In the previous study, selected isolates were additionally examined using the API Staph system (bioMérieux, France) and *rpoB* and *sodA* gene sequencing. Because 16 S rRNA sequencing may provide limited resolution for some closely related coagulase-negative staphylococci, this should be considered when interpreting species assignments.

In total, the 93 CoNS isolates represented 12 species (23 *S. cohnii*, 15 *S. epidermidis*, 14 *S. hominis*, 10 *Mammaliicoccus lentus*, 9 *S. saprophyticus*, 8 *S. chromogenes*, 4 *S. arlettae*, 4 *Mammaliicoccus sciuri*, 2 *S. haemolyticus*, 2 *S. xylosus*, 1 *S. carnosus*, and 1 *S. gallinarum*) and originated from multiple skeletal sites, including the femur, tibiotarsus, hock, stifle, footpad, and vertebrae [29]. *Mammaliicoccus sciuri* and *Mammaliicoccus lentus* (formerly *Staphylococcus sciuri and Staphylococcus lentus*, respectively) are referred to by their updated taxonomic names throughout the manuscript.

### Phenotypic assessment of antibiotic resistance

Antimicrobial susceptibility testing (AST) was performed by the disk diffusion method in accordance with the CLSI VET01 standard [30]. In short, overnight cultures of the bacteria were freshly prepared and suspended in 0.9% NaCl solution to achieve a turbidity equivalent to 0.5 on the McFarland scale using a DEN-1 densitometer (Biosan, Latvia). The bacterial suspensions were then inoculated onto Mueller-Hinton agar plates (GRASO Biotech, Poland) and antibiotic discs (listed in Supplementary Table S1) were evenly placed on the agar surface. Plates were incubated at 37 ± 1 °C for 18 ± 2 h under aerobic conditions. Following incubation, the inhibition zone diameters were measured in millimeters and interpreted according to CLSI veterinary clinical breakpoints. For florfenicol, because staphylococcal florfenicol-specific disk diffusion breakpoints are currently not available, inhibition zones were interpreted using CLSI chloramphenicol interpretive criteria as surrogate phenicol-class breakpoints. For organism-drug combinations (e.g., amoxicillin-clavulanate) without CLSI interpretive criteria, zone diameters were recorded without categorical interpretation. Quality control was performed in parallel using *Staphylococcus aureus* ATCC 25,923 [31].

Minimum inhibitory concentration (MIC) testing was carried out on a limited exploratory subset consisting of one randomly selected isolate from each of 10 of the 12 species identified in the collection, primarily to provide complementary descriptive data. The two remaining species, *S. carnosus* and *S. gallinarum*, were each represented by a single isolate and were not included in MIC testing. Commercially available MIC test strips (Liofilchem, Italy) containing a gradient of antibiotic concentrations were applied to the inoculated Mueller-Hinton agar plates, according to the manufacturer’s instructions. MIC values were read and interpreted using CLSI VET01S [31] criteria where applicable. Because only one isolate per species was tested, MIC data were used only as complementary quantitative results at the isolate level and were not intended for species-level inference.

### Detection of antibiotic resistance genes

Polymerase chain reaction (PCR) was employed to detect the presence of selected antibiotic resistance genes in the staphylococcal isolates. The following genes were targeted: *mecA* (methicillin resistance gene A), *mecC* (methicillin resistance gene C), *bla*_Z_ (β-lactamase gene), *erm*(A), *erm*(B), and *erm*(C) (erythromycin ribosomal methylase genes A, B, and C), *lnu*(A) (lincosamide nucleotidyltransferase gene A), *vga*(A) (virginiamycin A resistance gene), *tet*(K), *tet*(L), and *tet*(M) (tetracycline resistance genes K, L, and M), *fexA* (phenicol exporter gene A), *cfr* (multidrug resistance gene conferring resistance to phenicols, lincosamides, pleuromutilins, streptogramin A, and oxazolidinones), and *aac(6′)-Ie-aph(2″)-Ia* (aminoglycoside acetyltransferase/phosphotransferase gene).

Genomic DNA previously extracted from each isolate served as the template for the PCR assays. Reactions were performed using PCR Mix Plus Green (A&A Biotechnology, Poland). The original reference sources for the individual PCR protocols, primer sequences, and cycling conditions for each target gene are presented in Supplementary Table S2. Amplicons were visualized by electrophoresis in 1.5% agarose gels stained with ethidium bromide, and bands were compared to a molecular weight marker to confirm the expected product sizes.

### Statistical analysis

Statistical analyses were performed in R 4.3.0. Associations between binary and nominal variables were assessed using the chi‑squared test. Exploratory correlation analyses were performed to assess relationships between resistance genes, between phenotypic resistance profiles, and between genes and phenotypes. PCR-detected resistance genes were coded as binary variables (absence/presence: 0/1), whereas disk diffusion phenotypes were encoded ordinally as susceptible = 0, intermediate = 1, and resistant = 2. Isolates categorized as intermediate were retained as a separate interpretive category and were not automatically merged with either susceptible or resistant isolates. Gene-gene associations were assessed using Pearson correlation coefficients calculated on binary presence/absence data, whereas phenotype-phenotype and gene-phenotype associations were assessed using Spearman’s rank correlation. Each pairwise comparison was based on pairwise complete observations. For the gene-gene and gene-phenotype analyses, genes present in fewer than 3 isolates were excluded to reduce unstable estimates. P-values were adjusted for multiple testing using the Benjamini-Hochberg method, and only associations with adjusted *p* < 0.05 were displayed in the heatmaps. For analyses other than the corrected exploratory correlation screens, a p-value < 0.05 was considered statistically significant.

### Pulsed-field gel electrophoresis

Pulsed-field gel electrophoresis (PFGE) was performed on a subset of 21 *S. cohnii* isolates recovered from 9 broiler flocks. This species was selected because it was among the most frequently recovered species in the collection and included multiple isolates with multidrug-resistant phenotypes, allowing exploratory assessment of within-species genomic diversity. PFGE was conducted based on the harmonized protocol described by Murchan et al. (2003) [32], with minor modifications for coagulase-negative staphylococci. Overnight cultures in tryptic soy broth (TSB) were adjusted to a turbidity equivalent to 3.5 McFarland and mixed with 2% low-melting-point agarose (InCert, Lonza, USA) to prepare agarose discs. Lysis of bacterial cells in the plugs was performed at 37 °C for 18 h using lysostaphin (1 mg/mL, A&A Biotechnology, Poland), lysozyme (10 mg/mL, A&A Biotechnology, Poland), and RNase A (10 mg/mL, Thermo Fisher Scientific Inc., USA). Proteinase K (20 mg/mL, A&A Biotechnology, Poland) was then applied for overnight digestion at 50 °C. The plugs containing genomic DNA were digested overnight at 25 °C with SmaI restriction enzyme (20 U/µL, Thermo Fisher Scientific Inc., USA). Separation of restriction fragments was achieved using a CHEF-DR II system (Bio-Rad Laboratories Inc., USA) on 1% Pulsed Field Certified agarose (Bio-Rad Laboratories Inc., USA) in 0.5× TBE buffer at 14 °C, with pulse times ranging from 5 to 60 s for a total of 23 h. Gels were stained with ethidium bromide and visualized under UV light. Gel images were analyzed using Gel Compare II (Applied Maths, Belgium), and clustering was carried out using UPGMA with the Dice similarity coefficient (optimization 0.5%, position tolerance 1.5%). Isolates with ≥ 80% similarity were considered closely related and grouped into the same PFGE cluster, following the criteria of Tenover et al. (1995) [33].

### Whole genome sequencing of *Staphylococcus cohnii* strain SC27

Genomic DNA was extracted from the bacterial isolate *Staphylococcus cohnii* 27-0405LFP (hereafter referred to as SC27) and its quality was assessed prior to library preparation. DNA concentration was measured using the PicoGreen fluorometric assay (Life Technologies, P11496), with quantification performed on an Infinite plate reader (Tecan). The whole-genome shotgun project has been deposited in DDBJ/ENA/GenBank under accession JBSXGX000000000.

SC27 was selected on the basis of the data available at the time of selection, including its phenotypic and PCR-based resistance profile and its epidemiological context, specifically its recovery from a flock with a marked lameness problem and bone lesions. On the basis of these pre-WGS findings, SC27 was considered the most informative candidate for exploratory genomic characterization of resistance-associated elements within this species. This analysis was intended to be illustrative rather than population-representative.

#### Library preparation and sequencing

For Illumina MiSeq platform (Illumina, USA) sequencing, genomic DNA was fragmented by sonication using a Covaris E210 instrument (Covaris, USA), following the manufacturer’s recommended parameters for Illumina library preparation. Libraries were constructed using the NEBNext Ultra II DNA Library Prep Kit for Illumina (New England Biolabs, USA; E7645L) according to the supplier’s protocol.

For Oxford Nanopore sequencing, libraries were prepared with the Rapid Barcoding Kit (SQK-RBK004; Oxford Nanopore Technologies, UK) following the manufacturer’s instructions. Sequencing was performed on a MinION device (Oxford Nanopore Technologies, UK) using a SpotON Flow Cell Mk I (R9.4.1).

#### Bioinformatic analysis and functional annotation

Raw reads from the MiSeq platform were filtered using Cutadapt version 3.0 to remove adapters and low-quality sequences. Quality control of sequencing data was carried out with FastQC. Basecalling for Oxford Nanopore reads was performed using Guppy (version 6.1.2). De novo genome assembly was conducted with Unicycler version 0.4.7 (Wick et al., 2017). Assembly quality was additionally assessed using QUAST v5.3.0 [34] and BUSCO v6.0.0 with the *staphylococcaceae_odb12* lineage dataset [35].

Structural and functional annotation was performed at the Bacterial and Viral Bioinformatics Resource Center (BV-BRC) (bv-brc.org) using the RASTtk pipeline [36]. Annotation outputs were cross-referenced against domain databases as follows: virulence factors against VICTORS and Virulence Factor Database (VFDB), transporters against the Transporter Classification Database (TCDB), drug targets against DrugBank and the Therapeutic Target Database (TTD).

Antimicrobial resistance determinants were summarized from BV-BRC/PATRIC annotations and complemented by a targeted screen with Antimicrobial Resistance Identification By Assembly (ARIBA) [37] via Bactopia (version 2.1.0) using the NCBI Bacterial Antimicrobial Resistance Reference Gene Database. Results were adjusted at the gene symbol level to remove duplicates and synonyms prior to counting. This screening focused on acquired resistance genes. Systematic mutation-based resistance calling was not performed. Staphylococcal cassette chromosome *mec* (SCCmec) typing was carried out on the assembled chromosome with SCCmecFinder (CGE/DTU, version 1.2), and cassette recombinase loci were recorded together with percent identity and coverage as returned by the tool.

Plasmid mobility was assessed in silico using oriTfinder [38] by screening each plasmid contig for a putative origin of transfer (oriT) region and relaxase-associated transfer functions. The results were used descriptively to identify plasmids with putative mobilization potential. No experimental transfer assays were performed.

## Results

### PCR detection of resistance genes

Among the 93 CoNS isolates examined, the most frequently detected antimicrobial resistance genes by PCR were *bla*_Z_ and *lnu*(A) (each in 37.6%, n = 35 of isolates), followed by *tet*(L) (25.8%, n = 24); *tet*(M) (22.6%, n = 21), and *mecA* and *cfr* (each in 16.1%, n = 15). The *erm*(C) gene was found in 12.9% (n = 12) of isolates, while *erm*(A) and *erm*(B) were uncommon (2.2%, n = 2 and 1.1%, n = 1, respectively). Lower prevalence was noted for *vga*(A) (4.3%, n = 4), *fexA* (3.2% n = 3), and *tet*(K) (8.6%, n = 8). No isolates carried *mecC* or *aac(6′)-Ie-aph(2’’)-Ia*.

Gene distribution varied by species – for example, *bla*_Z_ was especially prevalent in *S. chromogenes* (87.5%, *n* = 7), *S. hominis* (78.6%, *n* = 11), and *S. epidermidis* (53.3%, *n* = 8), while *lnu*(A) was most frequent in *S. cohnii* (73.9%, *n* = 17). The highest proportion of *mecA*-positive isolates was detected in *S. cohnii* (30.4%, *n* = 7) and *S. hominis* (21.4%, *n* = 3). By lesion type, *bla*_Z_ was most common in isolates from femoral head necrosis (50.0%, *n* = 16) and abscesses (50.0%, *n* = 5), while *mecA* was most frequently detected in synovitis cases (41.7%, *n* = 5).

**Fig. 1:**
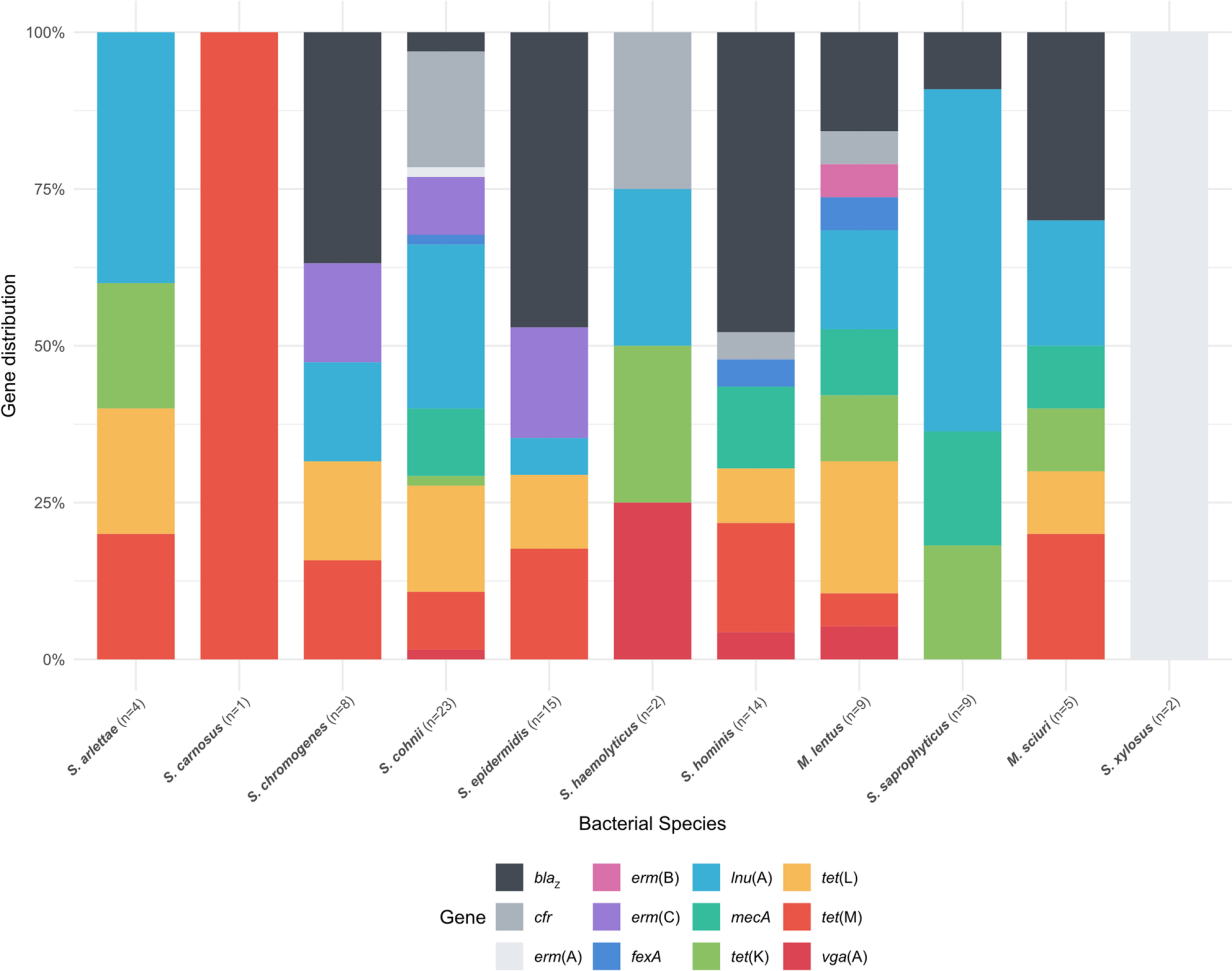
Distribution of PCR-detected antimicrobial-resistance genes by species. Stacked bars show the within-species distribution of PCR-detected resistance genes among coagulase-negative staphylococcal isolates. Species labels include the number of isolates in each group (n). For each species, the bar is normalized to 100%, and each segment represents the proportion of gene-positive detections attributed to a given gene within that species. Only genes included in the PCR screening panel are shown

A summary of gene detection frequencies is presented in Table 1. The distribution of resistance genes within species is shown in Fig. 1.

**Table 1:** Distribution of PCR-detected antimicrobial resistance genes found in *Staphylococcus* spp. isolates by lesion type

Lesion type	Total (*n*)	*mecA*	*mecC*	*bla*_*Z*_	*erm(A)*	*erm(B)*	*erm(C)*	*lnu(A)*	*vga(A)*	*tet(K)*	*tet(L)*	*tet(M)*	*fexA*	*cfr*	*aac(6′)-Ie-aph(2″)-Ia*
FHN	32	4 (12.5%)	0 (0.0%)	16 (50.0%)	2 (6.3%)	0 (0.0%)	3 (9.4%)	13 (40.6%)	4 (12.5%)	2 (6.3%)	7 (21.9%)	4 (12.5%)	3 (9.4%)	3 (9.4%)	0 (0.0%)
FHS	11	0 (0.0%)	0 (0.0%)	3 (27.3%)	0 (0.0%)	0 (0.0%)	1 (9.1%)	5 (45.5%)	0 (0.0%)	1 (9.1%)	3 (27.3%)	3 (27.3%)	0 (0.0%)	3 (27.3%)	0 (0.0%)
FHT	7	2 (28.6%)	0 (0.0%)	2 (28.6%)	0 (0.0%)	0 (0.0%)	3 (42.9%)	3 (42.9%)	0 (0.0%)	0 (0.0%)	2 (28.6%)	2 (28.6%)	0 (0.0%)	2 (28.6%)	0 (0.0%)
FPD	3	1 (33.3%)	0 (0.0%)	1 (33.3%)	0 (0.0%)	0 (0.0%)	0 (0.0%)	1 (33.3%)	0 (0.0%)	0 (0.0%)	1 (33.3%)	0 (0.0%)	0 (0.0%)	0 (0.0%)	0 (0.0%)
THN	5	1 (20.0%)	0 (0.0%)	3 (60.0%)	0 (0.0%)	0 (0.0%)	0 (0.0%)	0 (0.0%)	0 (0.0%)	1 (20.0%)	1 (20.0%)	1 (20.0%)	0 (0.0%)	0 (0.0%)	0 (0.0%)
Abscess	10	0 (0.0%)	0 (0.0%)	5 (50.0%)	0 (0.0%)	0 (0.0%)	0 (0.0%)	2 (20.0%)	0 (0.0%)	0 (0.0%)	0 (0.0%)	1 (10.0%)	0 (0.0%)	1 (10.0%)	0 (0.0%)
Necrosis	1	0 (0.0%)	0 (0.0%)	0 (0.0%)	0 (0.0%)	0 (0.0%)	0 (0.0%)	1 (100.0%)	0 (0.0%)	0 (0.0%)	0 (0.0%)	1 (100.0%)	0 (0.0%)	0 (0.0%)	0 (0.0%)
Normal	12	2 (16.7%)	0 (0.0%)	4 (33.3%)	0 (0.0%)	0 (0.0%)	2 (16.7%)	5 (41.7%)	0 (0.0%)	3 (25.0%)	2 (16.7%)	4 (33.3%)	0 (0.0%)	3 (25.0%)	0 (0.0%)
Synovitis	12	5 (41.7%)	0 (0.0%)	1 (8.3%)	0 (0.0%)	1 (8.3%)	3 (25.0%)	5 (41.7%)	0 (0.0%)	1 (8.3%)	7 (58.3%)	6 (50.0%)	0 (0.0%)	3 (25.0%)	0 (0.0%)
Sum / Overall	93	15 (16.1%)	0 (0.0%)	35 (37.6%)	2 (2.2%)	1 (1.1%)	12 (12.9%)	35 (37.6%)	4 (4.3%)	8 (8.6%)	24 (25.8%)	21 (22.6%)	3 (3.2%)	15 (16.1%)	0 (0.0%)

Values are given as n (%), where percentages were calculated within each lesion type. “Total (n)” indicates the number of isolates assigned to each lesion category

*Abbreviations and lesion categories: FHN* femoral head necrosis, *FHS* femoral head separation (epiphyseolysis), *FHT* femoral head transitional degeneration, *FPD* footpad dermatitis, *THN* tibial head necrosis, *Abscess* abscess localized at the free thoracic vertebra (FTV) or adjacent vertebrae, *Necrosis* BCO-like lesions found at sites other than those specified above, *Normal* no visible gross lesion, *BCO* bacterial chondronecrosis with osteomyelitis

### Phenotypic antibiotic susceptibility (disk diffusion)

Antibiotic susceptibility testing by disk diffusion showed high levels of resistance, with substantial variability between antibiotics (Table 2). Quality control results were within the acceptable CLSI ranges. For amoxicillin-clavulanate, no CLSI interpretive criteria were available. Therefore, zone diameters were recorded without categorical interpretation. Florfenicol screening results were interpreted cautiously using chloramphenicol criteria as surrogate phenicol-class breakpoints, as described in the Methods.

**Table 2:** Disk diffusion susceptibility profiles of *Staphylococcus* spp. isolates (*n* = 93) by antibiotic

Antibiotic (symbol)	Susceptible, *n* (%)	Intermediate, *n* (%)	Resistant, *n* (%)
Enrofloxacin (ENR)	42 (45.2%)	13 (14.0%)	38 (40.9%)
Erythromycin (E)	43 (46.2%)	7 (7.5%)	43 (46.2%)
Penicillin (P)	43 (46.2%)	0 (0.0%)	50 (53.8%)
Clindamycin (DA)	45 (48.4%)	21 (22.6%)	27 (29.0%)
Doxycycline (DO)	47 (50.5%)	3 (3.2%)	43 (46.2%)
Tetracycline (TE)	50 (53.8%)	0 (0.0%)	43 (46.2%)
Cefpodoxime (CPD)	58 (62.4%)	19 (20.4%)	16 (17.2%)
Cefoxitin (FOX)	77 (82.8%)	0 (0.0%)	16 (17.2%)
Trimethoprim-sulfamethoxazole (SXT)	81 (87.1%)	3 (3.2%)	9 (9.7%)
Florfenicol (FFC)	89 (95.7%)	0 (0.0%)	4 (4.3%)
Gentamicin (CN)	92 (98.9%)	1 (1.1%)	0 (0.0%)

Values are given as n (%), calculated from the full set of 93 isolates tested for each antimicrobial agent. Categories are based on disk diffusion interpretation as susceptible, intermediate, or resistant using CLSI veterinary interpretive criteria. For florfenicol, chloramphenicol interpretive criteria were used as surrogate phenicol-class breakpoints

The majority of isolates were susceptible to gentamicin (CN, 98.9%, *n* = 92), florfenicol (FFC, 95.7%, *n* = 89), and sulfamethoxazole-trimethoprim (SXT, 87.1%, *n* = 81). The highest resistance rates were observed for penicillin (P, 53.8%, *n* = 50), tetracycline (TE, 46.2%, *n* = 43), erythromycin (E, 46.2%, *n* = 43), and doxycycline (DO, 46.2%, *n* = 43), followed by enrofloxacin (ENR, 40.9%, *n* = 38) and clindamycin (DA, 29.0%, *n* = 27). Resistance to cefoxitin (FOX) and cefpodoxime (CPD) was detected in 17.2% (*n* = 16) of isolates in each case. Intermediate susceptibility was most frequent for clindamycin (DA, 22.6%, *n* = 21), cefpodoxime (CPD, 20.4%, *n* = 19), and enrofloxacin (ENR, 14.0%, *n* = 13). Full susceptibility and resistance profiles are shown in Table 2 and Supplementary Table S3. The frequency of resistant isolates by species and antibiotic is presented in Supplementary Table S4.

### Minimum inhibitory concentration testing

Exploratory MIC testing was performed on one randomly selected isolate each of 10 of the 12 species identified in the collection. Because of the very limited sample size, these data are presented descriptively and were not used to infer species-level susceptibility distributions. Across the tested subset, most isolates were susceptible to cefoxitin, cefpodoxime, gentamicin, and florfenicol. Resistance to tetracycline and doxycycline was observed in several species, and penicillin resistance was common in *S. epidermidis*, *M. lentus*, *S. saprophyticus*, *S. chromogenes*, *M. sciuri*, and *S. hominis*. Resistance to clindamycin, erythromycin, and enrofloxacin was observed in several isolates. Full MIC-based profiles are presented in Table 3.

**Table 3:** Minimum inhibitory concentrations (MICs) for representative coagulase-negative staphylococcal isolates and overall MIC distribution by antibiotic

Antibiotic	Representative isolate MIC (mg/L)										Overall distribution		
	*S. arlettae*	*S. haemolyticus*	*S. cohnii*	*S. epidermidis*	*M. lentus*	*S. hominis*	*S. saprophyticus*	*S. chromogenes*	*M. sciuri*	*S. xylosus*	MIC range	MIC_50_	MIC_90_
P	0.19 (I)	0.032 (S)	0.064 (S)	1 (R)	0.25 (R)	0.5 (R)	2 (R)	0.19 (R)	0.125 (R)	0.094 (S)	0.032–2	0.19	1
AMC	0.032 (I)	0.047 (S)	0.016 (S)	0.19 (S)	0.38 (S)	0.125 (S)	0.125 (S)	0.016 (S)	0.094 (S)	0.032 (S)	0.016–0.38	0.047	0.19
FOX	1.5 (S)	1 (S)	0.5 (S)	2 (S)	1 (S)	4 (S)	1.5 (S)	0.38 (S)	2 (S)	0.5 (S)	0.38–4	1	2
CPD	0.25 (S)	1.5 (S)	0.25 (S)	1 (S)	6 (I)	0.38 (S)	1.5 (S)	0.5 (S)	3 (I)	0.38 (S)	0.25–6	0.5	3
TE	96 (R)	12 (R)	32 (R)	0.38 (S)	32 (S)	0.19 (S)	48 (R)	48 (R)	32 (R)	0.75 (S)	0.19–96	32	48
DO	32 (R)	2 (R)	8 (R)	0.25 (I)	8 (R)	0.125 (S)	6 (R)	8 (R)	6 (R)	0.38 (I)	0.125–32	6	8
CN	0.047 (S)	0.094 (S)	0.016 (S)	0.25 (S)	0.047 (S)	0.094 (S)	0.023 (S)	0.047 (S)	0.047 (S)	0.032 (S)	0.016–0.25	0.047	0.094
DA	0.094 (S)	6 (R)	256 (R)	0.125 (S)	256 (R)	0.094 (S)	0.125 (S)	256 (R)	1 (I)	0.19 (S)	0.094–256	0.19	256
ENR	0.032 (S)	0.047 (S)	32 (R)	0.094 (S)	32 (R)	0.47 (S)	32 (R)	32 (R)	0.38 (S)	0.125 (S)	0.032–32	0.38	32
E	0.38 (S)	96 (R)	256 (R)	16 (R)	256 (R)	128 (R)	96 (R)	256 (R)	0.125 (S)	0.25 (S)	0.125–256	96	256
FFC	3 (S)	3 (S)	8 (I)	4 (S)	4 (S)	1.5 (S)	2 (S)	2 (S)	1.5 (S)	2 (S)	1.5–8	2	4
SXT	0.094 (S)	0.38 (S)	1.5 (S)	0.125 (S)	≤ 0.002 (R)	0.125 (S)	0.094 (S)	0.25 (S)	0.19 (S)	0.19 (S)	≤ 0.002–1.5	0.125	0.38

Values for individual species are MICs (mg/L) obtained for one randomly selected isolate from each tested species, with categorical interpretation in parentheses: S – susceptible, I – intermediate, R – resistant. Summary columns show the MIC range, MIC_50_, and MIC_90_ calculated across the tested isolates (*n* = 10 per antibiotic). MICs were determined by gradient diffusion strips

*Abbreviations: P* penicillin, *AMC* amoxicillin-clavulanate, *FOX* cefoxitin, *CPD* cefpodoxime, *TE* tetracycline, *DO* doxycycline, *CN* gentamicin, *DA* clindamycin, *ENR* enrofloxacin, *E* erythromycin, *FFC* florfenicol, *SXT* trimethoprim-sulfamethoxazole

### Exploratory correlation analyses

**Fig. 2:**
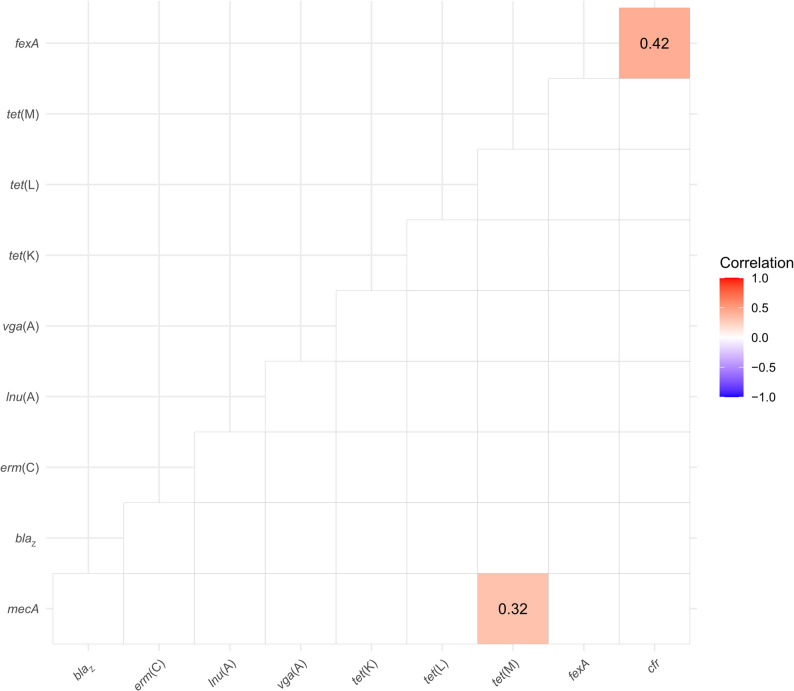
Exploratory gene-gene association heatmap. Pairwise correlations between PCR-detected resistance genes coded as binary presence/absence variables (0/1). Numbers in the cells indicate correlation coefficients, and blank cells indicate associations that were not significant after Benjamini-Hochberg correction. Genes present in fewer than 3 isolates were excluded from the analysis

**Fig. 3:**
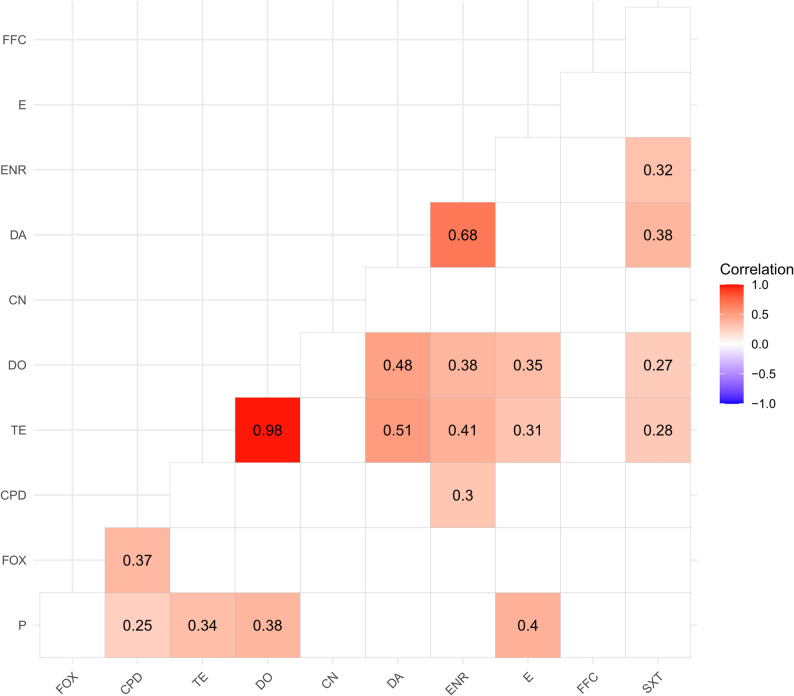
Exploratory phenotype-phenotype association heatmap based on disk diffusion data. Pairwise Spearman correlations between disk-diffusion phenotypes encoded ordinally as susceptible = 0, intermediate = 1, and resistant = 2. Numbers in the cells indicate correlation coefficients, and blank cells indicate associations that were not significant after Benjamini-Hochberg correction

**Fig. 4:**
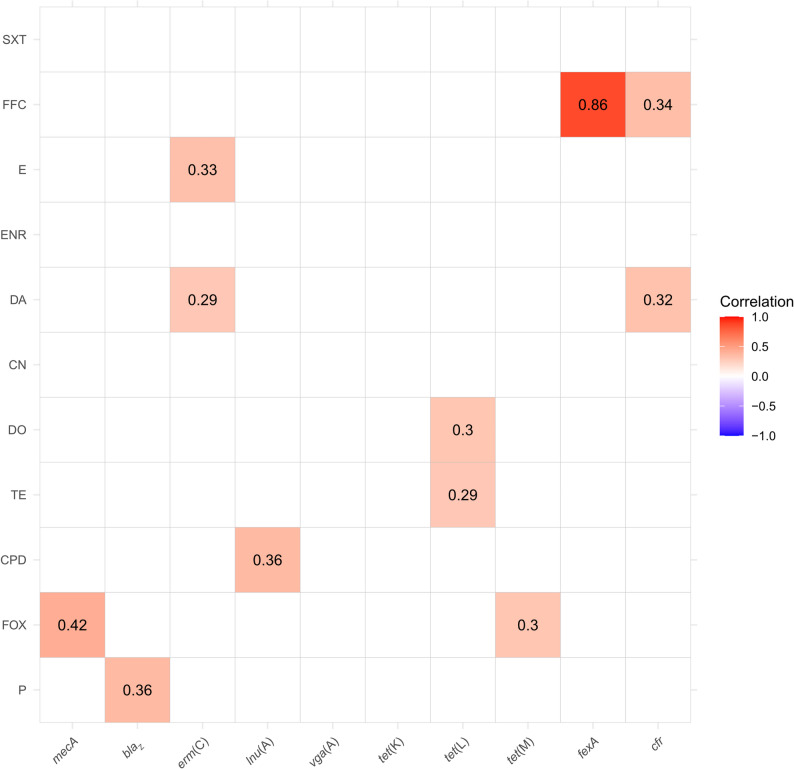
Exploratory gene-phenotype association heatmap based on disk diffusion data. Pairwise Spearman correlations between PCR-detected resistance genes coded as binary presence/absence variables (0/1) and disk-diffusion phenotypes encoded ordinally as susceptible = 0, intermediate = 1, and resistant = 2. Numbers in the cells indicate correlation coefficients, and blank cells indicate associations that were not significant after Benjamini-Hochberg correction. Genes present in fewer than 3 isolates were excluded from the analysis

To assess how clearly the targeted resistance genes corresponded to the observed resistance phenotypes in this dataset, and to identify broader co-resistance patterns or genotype-phenotype mismatches, we generated three complementary heatmaps showing gene-gene, phenotype-phenotype, and gene-phenotype associations (Figs. 2, 3 and 4). After correction for multiple testing, the gene-gene analysis identified only two significant positive correlations, namely *fexA*-*cfr* (Pearson’s *r* = 0.42, adjusted *p* < 0.05) and *mecA*-*tet*(M) (*r* = 0.32, adjusted *p* < 0.05) (Fig. 2). The phenotype-phenotype analysis showed several positive correlations, the strongest of which was between tetracycline (TE) and doxycycline (DO) (Spearman’s rho = 0.98, adjusted *p* < 0.05). Additional moderate associations that remained significant after correction were observed for clindamycin (DA)-enrofloxacin (ENR), tetracycline (TE)-clindamycin (DA), doxycycline (DO)-clindamycin (DA), tetracycline (TE)-enrofloxacin (ENR), penicillin (P)-erythromycin (E), penicillin (P)-doxycycline (DO), doxycycline (DO)-enrofloxacin (ENR), clindamycin (DA)-sulfamethoxazole-trimethoprim (SXT), and cefoxitin (FOX)-cefpodoxime (CPD) (Fig. 3). In the gene-phenotype analysis, the strongest association was observed between florfenicol (FFC) and *fexA* (rho = 0.86, adjusted *p* < 0.05), while more moderate associations that remained significant after correction were detected for cefoxitin (FOX)-*mecA*, penicillin (P)-*bla*_Z_, cefpodoxime (CPD)-*lnu*(A), *florfenicol* (FFC)*-cfr*, erythromycin (E)-*erm*(C), clindamycin (DA)-*erm*(C), clindamycin (DA)-*cfr*, doxycycline (DO)-*tet*(L), tetracycline (TE)-*tet*(L), and cefoxitin (FOX)-*tet*(M) (Fig. 4).

### Pulsed-field gel electrophoresis

**Fig. 5:**
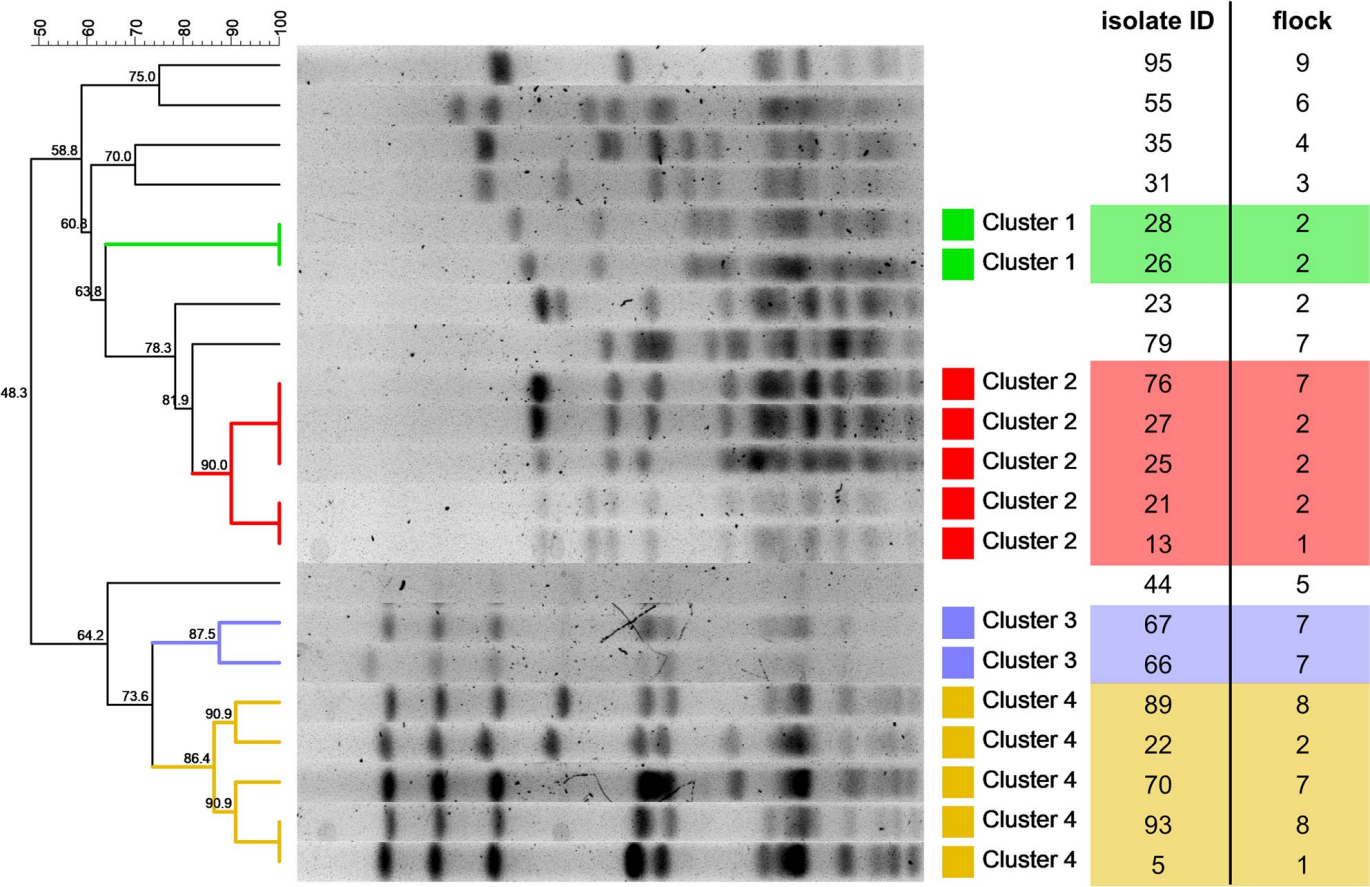
PFGE of *Staphylococcus cohnii* isolates with dendrogram and cluster labels. SmaI macrorestriction patterns of 21 *Staphylococcus cohnii* isolates from 9 broiler flocks are shown together with the corresponding UPGMA dendrogram based on the Dice similarity coefficient. Colored brackets indicate clusters defined at the preset similarity threshold of ≥ 80%. Isolates not included within the colored brackets were treated as singletons. The right-hand panel shows cluster assignment together with isolate IDs and flock IDs

PFGE of 21 *S. cohnii* isolates from 9 broiler flocks, using a similarity cut-off ≥ 80%, resolved 4 clusters (C1–C4) comprising 14/21 isolates. Two clusters contained 2 isolates each and two clusters contained 5 isolates each, and 7 isolates were singletons. The detected clusters were distributed across multiple flocks, and clustered isolates were recovered from several lesion categories, including BCO-spectrum lesions (FHT, FHN, THN). The full isolate-to-flock and lesion mapping is shown in Fig. 5.

### Whole-genome sequencing and genomic features of *Staphylococcus cohnii* SC27

#### Genome overview

Whole genome sequencing of *Staphylococcus cohnii* SC27 yielded a high-quality draft genome (8 contigs, total 2,705,049 bp, GC content 32.44%). The assembler output indicated one chromosomal contig (2,616,820 bp) and seven plasmid contigs, all flagged as circular. A total of 2,576 coding sequences (CDS) were annotated, including 531 hypothetical proteins. Detailed assembly quality metrics are provided in Supplementary Table S5.

#### Mobile genetic elements and resistome

After removing duplicates and considering synonyms, 19 named or clearly resistance-associated determinants were identified in SC27. These included genes associated with resistance to aminoglycosides, β-lactams, fluoroquinolones, fosfomycin, macrolides, lincosamides, tetracyclines, phenicols, and trimethoprim. The complete list of resistance-associated genes (including a broader set of target-associated, regulatory, efflux, and cell-envelope-related resistance annotations) identified by BV-BRC/PATRIC and ARIBA is presented in Supplementary Table S6.

BLASTn analysis showed that the plasmid contigs were highly similar to previously described staphylococcal plasmids from various species (including *S. aureus*, *S. saprophyticus*, *S. epidermidis* and *S. cohnii*). The plasmids together carried several antibiotic-resistance genes (Supplementary Table S7): pSC27_46k encoded *tet*(L), *fosB* (fosfomycin resistance), and *dfrK* (trimethoprim resistance); pSC27_3k90 carried *ant*(9)*-I* (spectinomycin nucleotidyltransferase); pSC27_2k46 encoded a small multidrug-resistance (SMR) family efflux transporter linked to biocide and quaternary ammonium cations tolerance; pSC27_2k36 harbored *erm*(C) (MLS_B_); pSC27_2k35 carried *lnu*(A) (lincosamide nucleotidyltransferase). No canonical AMR genes were detected on pSC27_29k or pSC27_1k38.

SCCmecFinder identified cassette chromosome recombinase genes *ccrB3* (86.6% identity) and *ccrA1* (83.7% identity) on the same chromosome, consistent with SCCmec-like element in *S. cohnii* SC27.

oriTfinder analysis did not identify any SC27 plasmid as self-transmissible. The largest plasmid, pSC27_46k, carried a putative oriT region and a putative relaxase with a Mob_Pre domain, which is consistent with a putatively mobilizable plasmid. Plasmid pSC27_3k90 yielded a putative relaxase-like hit with a Rep_trans domain but no detectable oriT region and was therefore not classified as mobilizable. No transfer-related features were detected in the remaining plasmids in this screen.

Overall, the genomic findings in SC27 did not always correspond to the observed phenotypic resistance profile. The most consistent matches were seen for tetracycline and erythromycin/clindamycin resistance, in line with the presence of *tet*(L), *erm*(C), and *lnu*(A). In contrast, *cfr* was detected despite phenotypic florfenicol susceptibility, and *mecA* detection was not supported by phenotypic testing with cefoxitin and the other tested β-lactams.

#### Virulence factors

*S. cohnii* SC27’s genome harbored classical staphylococcal virulence determinants (Supplementary Table S8). These included stress response and immune evasion proteins (ClpX, ClpP, RecA, CitB), cell wall synthesis (FemB), global regulators (CcpA, MgrA), metabolic enzymes (TrpB, PurL), adherence factors (OppD), iron uptake systems (IscR), and capsule and degradative enzymes (Cap8E, Cap8G). Additional transporter- and drug-target-related annotations identified in SC27 are presented in Supplementary Table S9.

## Discussion

This study provides phenotypic and genotypic characterization of AMR among CoNS isolated from skeletal lesions in broiler chickens. The results showed frequent resistance to penicillin, tetracycline/doxycycline, and erythromycin, together with frequent detection of *bla*_Z_, *lnu*(A), *tet*(L), *tet*(M), and *erm*(C). They also showed incomplete agreement between *mecA* carriage and phenotypic β-lactam resistance, genetic heterogeneity within the analyzed *S. cohnii* subset by PFGE, and isolate-specific resistance-associated elements in *S. cohnii* SC27 identified by WGS.

The detection of the *mecA* gene in 16.1% of isolates, primarily in *S. cohnii* and *S. hominis*, confirms the presence of methicillin resistance among poultry-associated CoNS. This is consistent with previous reports indicating that CoNS from poultry may carry clinically relevant resistance determinants and may contribute to the broader epidemiology of antimicrobial resistance in animal production systems [39, 40].

At the same time, the present results also show that the relationship between genotype and phenotype was not always straightforward. In particular, *mecA* was detected in a subset of isolates that were not consistently phenotypically resistant to cefoxitin or other β-lactam antibiotics. In our collection, this *mecA*-positive but cefoxitin-susceptible pattern was observed in three *S. cohnii* isolates, two *S. saprophyticus* isolates, one *S. hominis* isolate, and one *M. lentus* isolate. These findings indicate that cefoxitin disk diffusion, although useful as a routine screening approach, did not fully reflect *mecA* carriage in all represented CoNS species. This pattern is consistent with previous reports showing that heterogeneous expression of methicillin resistance may complicate phenotypic detection [41]. Similar findings have been described in both *S. aureus* and CoNS, where *mecA*-positive isolates may remain phenotypically susceptible under routine testing conditions or show only low-level expression of resistance [42–45]. In a recent study on CoNS, oxacillin-susceptible *mecA*-positive isolates were identified among species including *S. saprophyticus* and *S. epidermidis*, and cefoxitin disk diffusion showed lower sensitivity than oxacillin-based methods [46]. Likewise, in *S. lugdunensis*, cefoxitin disk diffusion and MIC-based interpretation failed to detect a subset of *mecA*-positive isolates, which were shown to display heterogeneous oxacillin resistance with resistant subpopulations at low frequencies [47]. These discrepancies may result from low expression of *mecA*, mutations in regulatory genes (e.g., *femA*,* femC*), the presence of non-functional *mec* gene cassettes, heteroresistance, species-specific differences in resistance expression, or limitations of routine phenotypic testing [41, 42, 45, 47]. Because no confirmatory assays such as PBP2a detection, expression analysis, or detailed cassette characterization were performed in the present study, these possibilities remain unresolved.

In our collection, phenotypic resistance to β-lactams was more frequent than the *mecA* carriage alone would suggest. This may be partly explained by the presence of *bla*_Z_, which was detected in 27 of the 50 penicillin-resistant isolates. The gene encodes the staphylococcal β-lactamase, a well-recognized determinant of penicillin resistance in staphylococci from both human and animal sources [39, 40, 48, 49].

The frequent detection of *bla*_Z_, *lnu*(A), *tet*(L), *tet*(M), and *erm*(C) in the present study suggests that resistance in these bacteria is driven by multiple mechanisms rather than by a single dominant determinant. A similar coexistence of tetracycline- and macrolide/lincosamide-associated genes has been previously reported in poultry-associated staphylococci, including isolates from Poland and Denmark [39, 40]. The presence of *cfr* in 16.1% of the isolates is also noteworthy, given that this gene has also been described in methicillin-resistant CoNS from poultry and other food animals in China, where it was associated with mobile genetic elements that may facilitate its dissemination [50–52]. At the same time, *cfr* carriage should not be automatically equated with florfenicol resistance. In our dataset, florfenicol showed a more consistent association with *fexA* than with *cfr*, and *cfr* was also detected in florfenicol-susceptible isolates. Similar genotype-phenotype mismatch has been described in staphylococci, including *cfr*-positive but phenotypically susceptible isolates in which a single Q148K substitution in Cfr prevent expression of the expected PhLOPS_A_ (phenicols, lincosamides, oxazolidinones, pleuromutilins, and streptogramin A) resistance phenotype [53]. These observations suggest that detection of *cfr* alone may overestimate phenicol resistance when gene functionality or phenotypic expression is not confirmed.

Although antimicrobial usage was not assessed directly in this study, the diversity of resistance genes identified in the analyzed isolates may reflect long-term or repeated exposure to different antimicrobial classes in poultry production. Similar observations have been reported in China, where the occurrence of genes such as *cfr*, *fexA*, *erm*(C), and *tet*(L) in poultry-associated staphylococci was linked to historical antimicrobial treatment data from poultry farms [50]. The present study was based on isolates recovered from 25 commercial broiler flocks predominantly in eastern Poland. The findings should be interpreted in relation to this geographic area and production setting and should not be considered representative of all broiler production systems or AMR patterns in poultry more broadly.

High resistance levels to penicillin (53.8%), doxycycline (46.2%), and erythromycin (46.2%) are in agreement with European and Asian poultry data. For example, Hungarian studies have shown doxycycline resistance in 74.4% of *S. aureus* isolates, and enrofloxacin resistance exceeding 50% in broilers [54]. Resistance patterns in Danish poultry isolates showed similarities to our findings [40], while data from China highlighted the widespread occurrence of the *cfr* gene in both retail meat and live animals [50, 51].

One of the key findings is that CoNS isolated from the femoral head lesions (FHN/FHT/FHS; *n* = 50) harbored multiple resistance genes including *bla*_Z_ and *lnu*(A) (each detected in 42.0% of isolates), with additional contributions from *tet*(L) (24.0%), *tet*(M) (18.0%), *cfr* (16.0%), and *mecA* (12.0%). Although these findings indicate that isolates recovered from femoral-head lesions can carry a broad range of resistance determinants, they should not be interpreted as evidence that this lesion type uniquely concentrates AMR genes. Rather, the resistance profile observed in femoral head lesions broadly mirrored the wider dataset. At the same time, the present findings should not be interpreted as support for routine antimicrobial susceptibility testing of any CoNS recovered from skeletal lesions without appropriate pathological and microbiological context.

BLASTn analysis showed that the SC27 plasmids were highly similar to previously described plasmids from several *Staphylococcus* species. This supports the mosaic character of staphylococcal plasmids and their role in assembling combinations of resistance genes. At the same time, sequence similarity alone does not demonstrate active interspecies plasmid exchange, and the present WGS results should therefore be interpreted as isolate-specific rather than as direct evidence of ongoing transfer. This more cautious interpretation is also consistent with recent work showing that genes such as *cfr* and *fexA* may occur across human and livestock-associated staphylococci, while direct spread of a specific plasmid or clone is not always demonstrable [51, 52, 55].

Whole-genome sequencing of *S. cohnii* SC27 identified multiple plasmid-borne resistance genes. Several of the detected genes, including *bla*, *erm*(C), *fexA*, and *dfrK*, have been previously associated with mobile genetic elements such as plasmids, transposons, and SCCmec cassettes [48, 51, 52]. One notable finding was the presence of *tet*(L), *fosB*, and *dfrK* on plasmid pSC27_46k. Kadlec and Schwarz [56] reported a closely related *tet*(L)-*dfrK* linkage in a multiresistance plasmid from MRSA ST398, with a 282-bp spacer separating the two genes. The same 282-bp intergenic distance was observed in pSC27_46k, indicating a highly similar genetic arrangement. This conserved organization suggests that these genes may be maintained together and co-selected under antimicrobial pressure.

SCCmecFinder additionally detected the cassette chromosome recombinase genes *ccrA1* and *ccrB3*, consistent with the presence of an SCCmec-like element in SC27. This observation is relevant because methicillin resistance genes and associated recombinase systems are central to the mobility and evolution of β-lactam resistance within staphylococci [49]. However, the present data do not allow reconstruction of the complete cassette structure or determination of its full gene content.

The plasmid mobility screen suggested limited transfer potential among the SC27 plasmids. None of the plasmids showed a complete set of transfer-related modules expected for a self-transmissible conjugative element. The largest plasmid, pSC27_46k, carried both a putative oriT region and a relaxase with a Mob_Pre domain, supporting its interpretation as a putatively mobilizable plasmid. By contrast, pSC27_3k90 showed only a weaker putative relaxase-like hit with a Rep_trans domain and lacked a detectable oriT region, so it was not classified as mobilizable. These findings suggest that some plasmids may be mobilizable, although active conjugative transfer was not demonstrated in the present study [57, 58].

Figures 2, 3 and 4 show exploratory analyses of associations between resistance genes, resistance phenotypes, and gene-phenotype pairs. The aim of these analyses was not simply to restate expected resistance mechanisms, but to assess how well the targeted PCR panel reflected the observed phenotypic patterns in this collection and to identify broader co-resistance patterns or cases in which genotype and phenotype did not fully match. In the gene-gene analysis, only two associations remained significant after Benjamini-Hochberg correction: *fexA*-*cfr* and *mecA*-*tet*(M). This indicates that repeated co-occurrence of resistance genes was limited in the analyzed isolates. The *fexA*-*cfr* association is plausible because both genes have previously been reported together in transferable resistance elements [53, 59], while the weaker *mecA*-*tet*(M) association may simply reflect their co-occurrence in multidrug-resistant isolates [60, 61].

The phenotype-phenotype analysis showed several significant positive correlations. The strongest was the tetracycline-doxycycline association, which was expected because both agents belong to the same antimicrobial class and often share resistance mechanisms [62]. Other moderate correlations, including clindamycin-enrofloxacin, tetracycline-clindamycin, penicillin-erythromycin, and cefoxitin-cefpodoxime, are more likely to reflect co-resistance within the same isolates than any specific relationship between the drugs themselves [24, 63].

In the gene-phenotype analysis, the strongest and most biologically plausible association was florfenicol-*fexA*. Moderate associations were also observed for cefoxitin-*mecA*, penicillin-*bla*_Z_, erythromycin/clindamycin-*erm*(C), and tetracycline/doxycycline-*tet*(L), which are generally consistent with expected resistance mechanisms [64–66]. At the same time, not all gene-phenotype relationships were equally clear. In particular, florfenicol showed a more consistent association with *fexA* than with *cfr*, and *mecA* showed only partial correspondence with cefoxitin resistance, consistent with the broader genotype-phenotype patterns discussed above. Other significant associations were weaker and are more difficult to interpret directly. They may reflect co-occurrence of genes in the same isolates, the presence of genes on the same mobile elements, or the fact that some isolates carried several resistance determinants at the same time [24, 67, 68]. Overall, these heatmaps should be treated as exploratory statistical summaries rather than as evidence of direct causal relationships.

The PFGE analysis of 21 *S. cohnii* isolates showed that clustered and singleton pulsotypes co-occurred within the analyzed subset, indicating that more than one closely related genomic background was present among the studied isolates. Because some clusters were represented in more than one flock, whereas others were restricted to a single flock, the PFGE data are compatible with genetic heterogeneity within this species. PFGE at the level used here does not provide sufficient evidence to infer transmission routes, repeated introductions, or persistent environmental reservoirs. A similar caution is warranted in light of previous work on *cfr*-positive methicillin-resistant CoNS from food animals, in which diversity of PFGE patterns was taken to support horizontal dissemination of resistance genes rather than simple clonal expansion [50].

Although no hypervirulence-associated pattern was observed, the SC27 genome contained several genes related to stress response, regulation, adhesion, iron acquisition, and persistence. This is compatible with the established view that CoNS should not be regarded as uniformly harmless commensals, but rather as bacteria with context-dependent opportunistic pathogenic potential [39, 69]. The present study did not include a dedicated comparative virulence analysis, and the detected genes should therefore be interpreted as supportive context rather than proof of a specific lesion-causing mechanism.

The overlap between resistance determinants found in poultry-associated staphylococci and those reported in human-, livestock-, and companion animal-associated isolates supports the broader One Health relevance of these findings. Genes such as *mecA*, *cfr*, *fexA*, *erm*(C), and tetracycline resistance determinants have been documented in poultry, livestock, retail meat, and human-associated staphylococci, and veterinary clinical isolates from companion animals [50, 51, 55, 70, 71]. However, our data do not directly demonstrate zoonotic transmission, food-chain transfer, or human infection risk. For this reason, the One Health significance of the present findings is best framed in terms of resistance surveillance and reservoir potential rather than direct evidence of cross-sector spread.

## Conclusions

CoNS recovered from bone and joint lesions of lame broiler chickens showed diverse phenotypic and genotypic antimicrobial resistance profiles, with frequent resistance to penicillin, tetracycline/doxycycline, and erythromycin, and frequent detection of *bla*_Z_, *lnu*(A), *tet*(L), *tet*(M), and *erm*(C). Together, these findings indicate that CoNS recovered from skeletal lesions in broiler chickens may represent an important reservoir of antimicrobial resistance determinants within poultry production systems. The study also adds new genomic and epidemiological context to current knowledge of AMR in broiler-associated CoNS and supports their inclusion in ongoing veterinary and One Health-oriented surveillance efforts.

## Supplementary Information

Supplementary Material 1.

Supplementary Material 2.

## Data Availability

Supporting data are presented in the article and the Supplementary Materials. The whole-genome shotgun sequence of *Staphylococcus cohnii* 27-0405LFP has been deposited in DDBJ/ENA/GenBank under accession JBSXGX000000000. The version described in this paper is JBSXGX010000000.

## Abbreviations

16S rRNA: 16 S ribosomal ribonucleic acid
AMR: Antimicrobial resistance
AMC: Amoxicillin–clavulanate
ARIBA: Antimicrobial Resistance Identification By Assembly
AST: Antimicrobial susceptibility testing
BCO: Bacterial chondronecrosis with osteomyelitis
BLASTn: Basic Local Alignment Search Tool for nucleotides
bp: Base pair(s)
BUSCO: Benchmarking Universal Single–Copy Orthologs
BV: BRC–Bacterial and Viral Bioinformatics Resource Center
CDS: Coding sequences
CLSI: Clinical and Laboratory Standards Institute
CN: Gentamicin
CoNS: Coagulase–negative staphylococci
CPD: Cefpodoxime
DA: Clindamycin
DD: Disk diffusion
DNA: Deoxyribonucleic acid
DO: Doxycycline
E: Erythromycin
ENR: Enrofloxacin
FFC: Florfenicol
FHN: Femoral head necrosis
FHS: Femoral head separation
FHT: Femoral head transitional degeneration
MDR: Multidrug–resistant
MIC: Minimum inhibitory concentration
MLS_B_: Macrolide–lincosamide–streptogramin B
MRCoNS: Methicillin–resistant coagulase–negative staphylococci
MRSA: Methicillin–resistant Staphylococcus aureus
MRSE: Methicillin–resistant Staphylococcus epidermidis
NCBI: National Center for Biotechnology Information
P: Penicillin
PCR: Polymerase chain reaction
PhLOPS_A_: Phenicols, lincosamides, oxazolidinones, pleuromutilins, and streptogramin A
PFGE: Pulsed–field gel electrophoresis
QUAST: Quality Assessment Tool for Genome Assemblies
RASTtk: Rapid Annotations using Subsystems Technology toolkit
SCCmec: Staphylococcal cassette chromosome mec
SMR: Small multidrug–resistance
SXT: Sulfamethoxazole–trimethoprim
TCDB: Transporter Classification Database
TE: Tetracycline
TBE: Tris–borate–EDTA buffer
THN: Tibial head necrosis
TSB: Tryptic soy broth
TTD: Therapeutic Target Database
UPGMA: Unweighted pair group method with arithmetic mean
VFDB: Virulence Factor Database
VICTORS: Virulence Factors of Pathogenic Bacteria database
VME: Very major error
WGS: Whole–genome sequencing
