# Supplementary material for: Phenotypic and genotypic characterization of antimicrobial resistance in coagulase-negative staphylococci from bone lesions in broiler chickens
Source: BMC Vet Res. 2026 May 26;22:435. doi: 10.1186/s12917-026-05584-8 (PMC13393699; doi:10.1186/s12917-026-05584-8)
Supplement: Supplementary file 1 — Supplementary Material 1. [file 12917_2026_5584_MOESM1_ESM.docx]

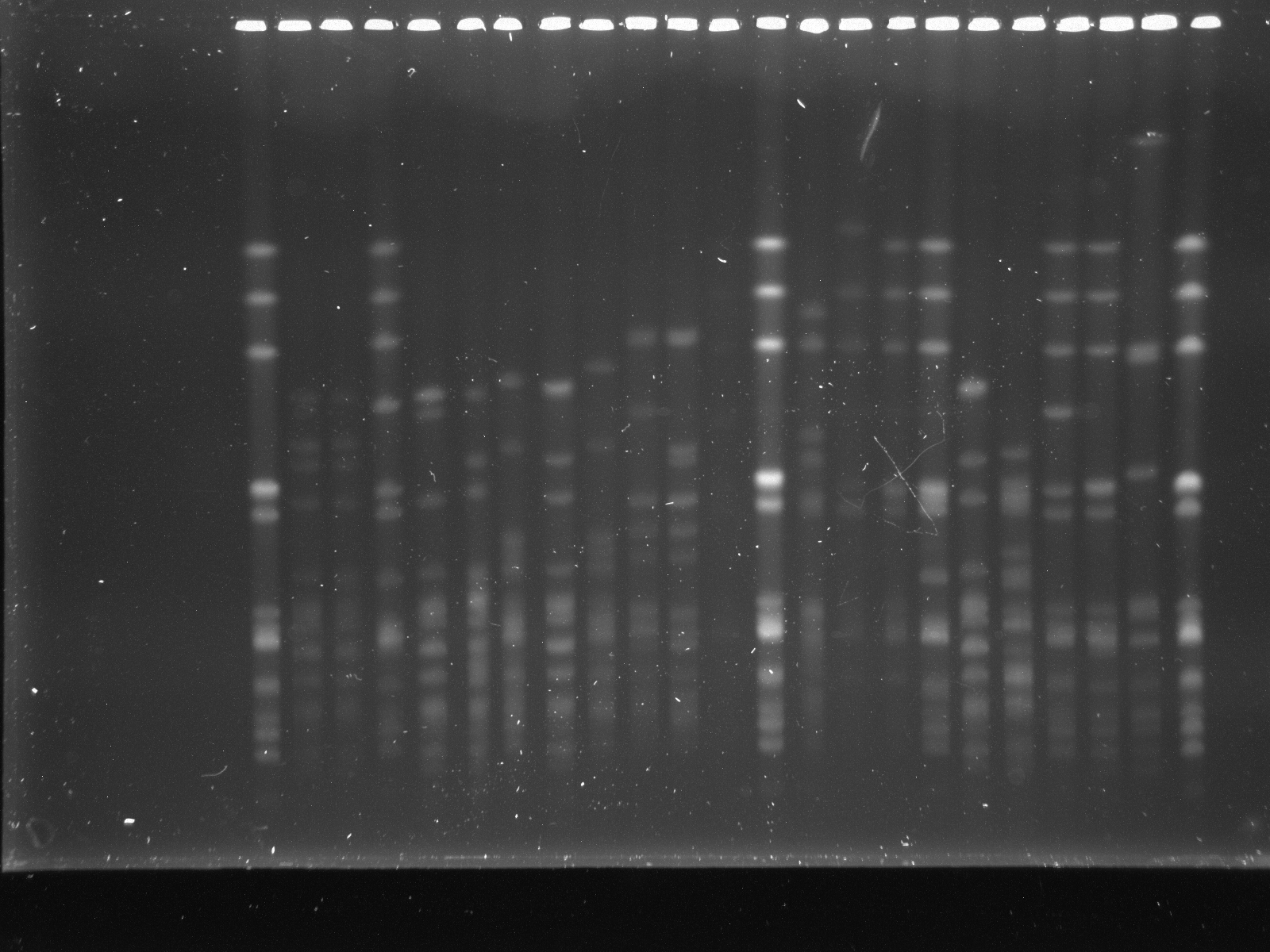


Figure S1. Original uncropped pulsed-field gel electrophoresis (PFGE) image corresponding to Figure 1 in the main text.


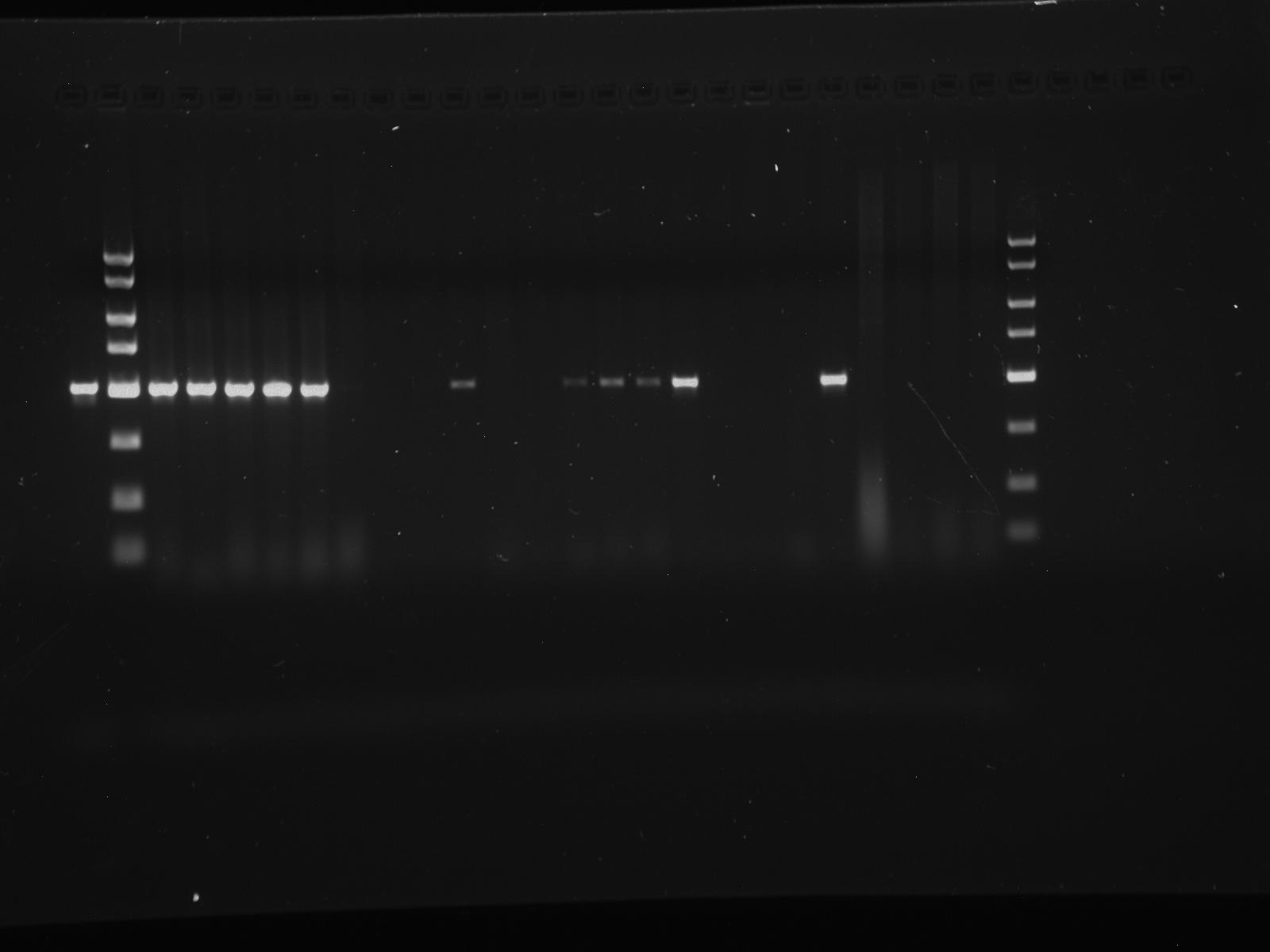


A.


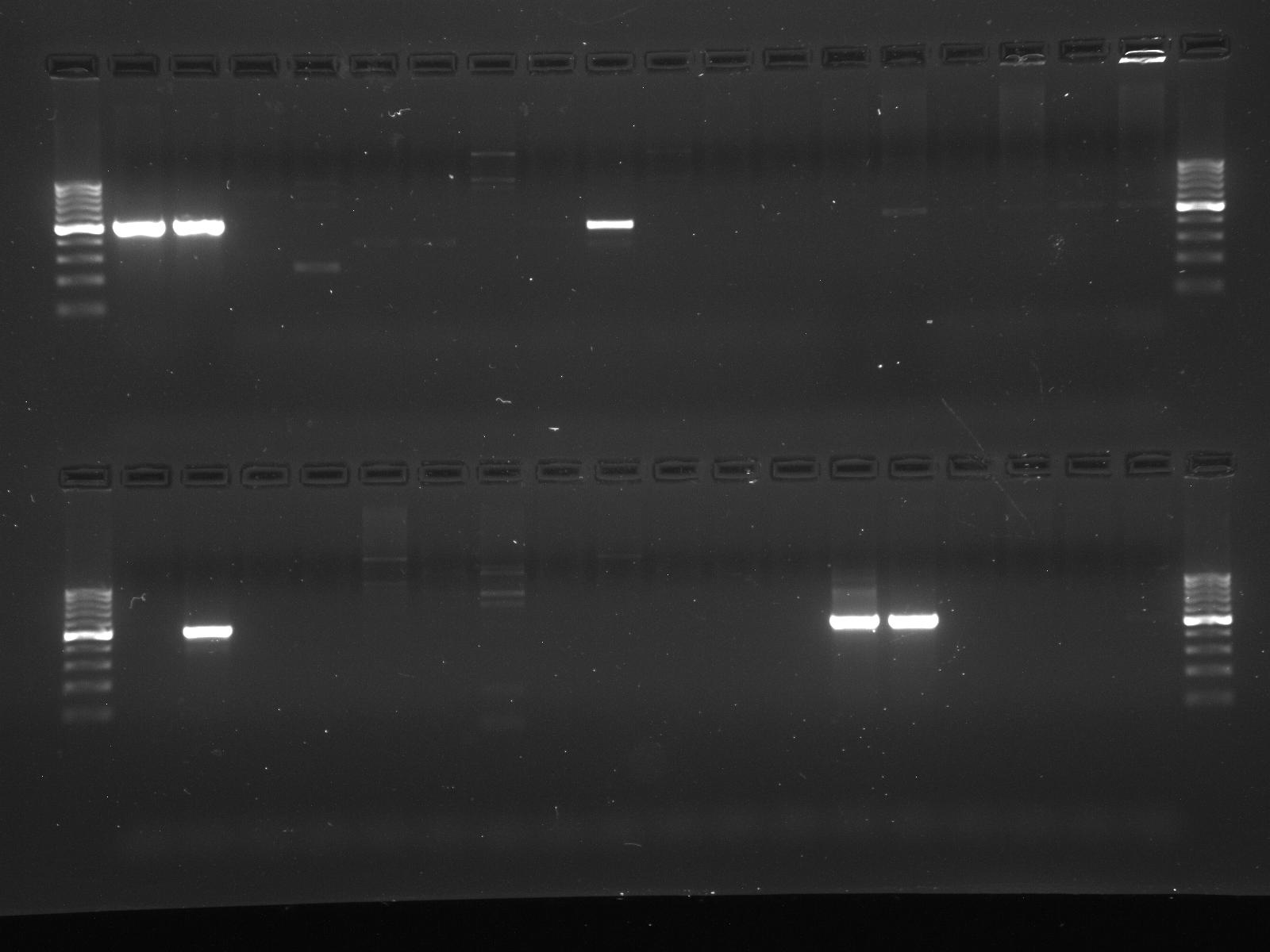


B.


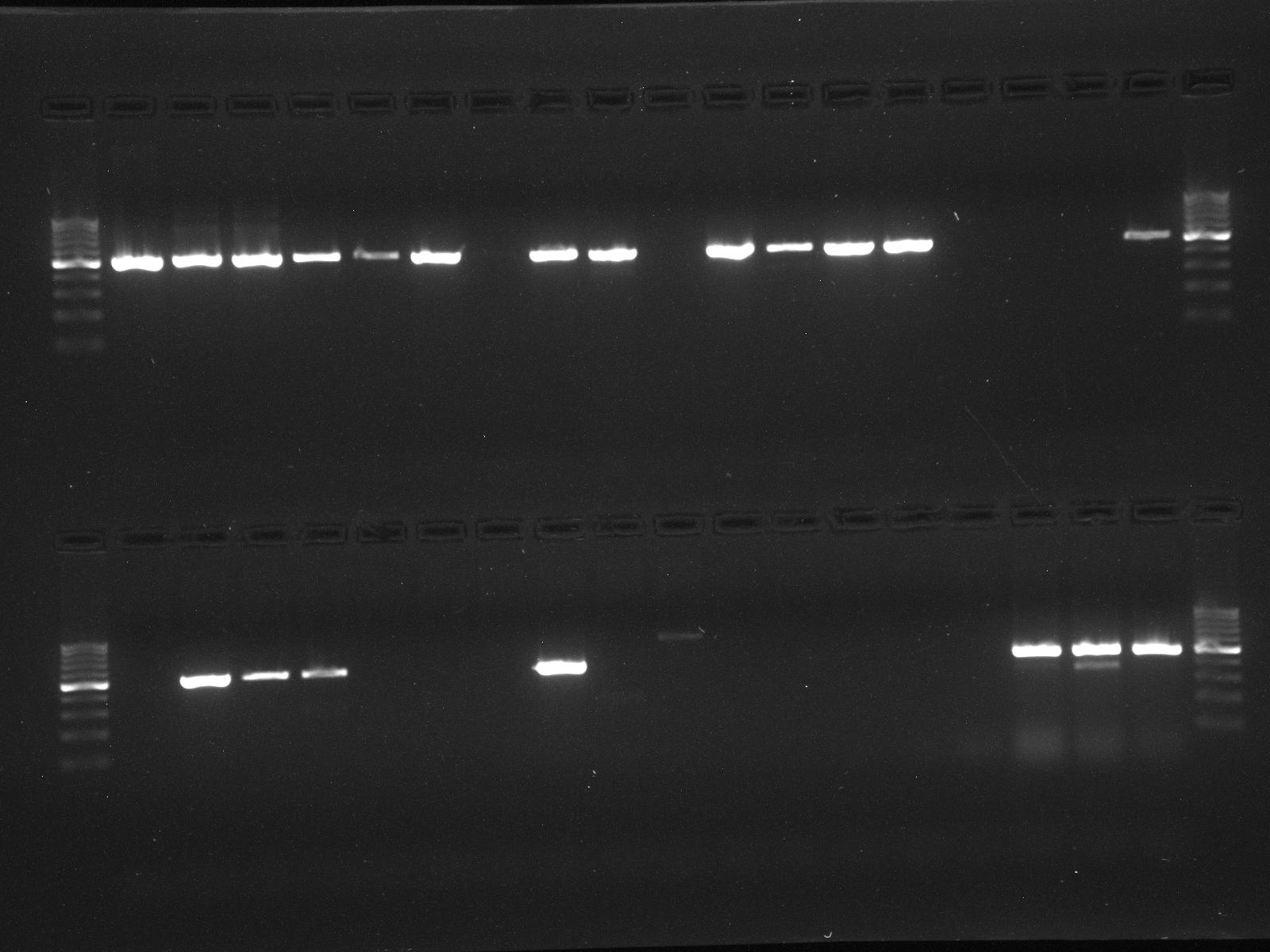


C.

Figure S2 (A, B, C). Agarose gel electrophoresis of amplified PCR products of the *blaZ* gene (530 bp amplicon).


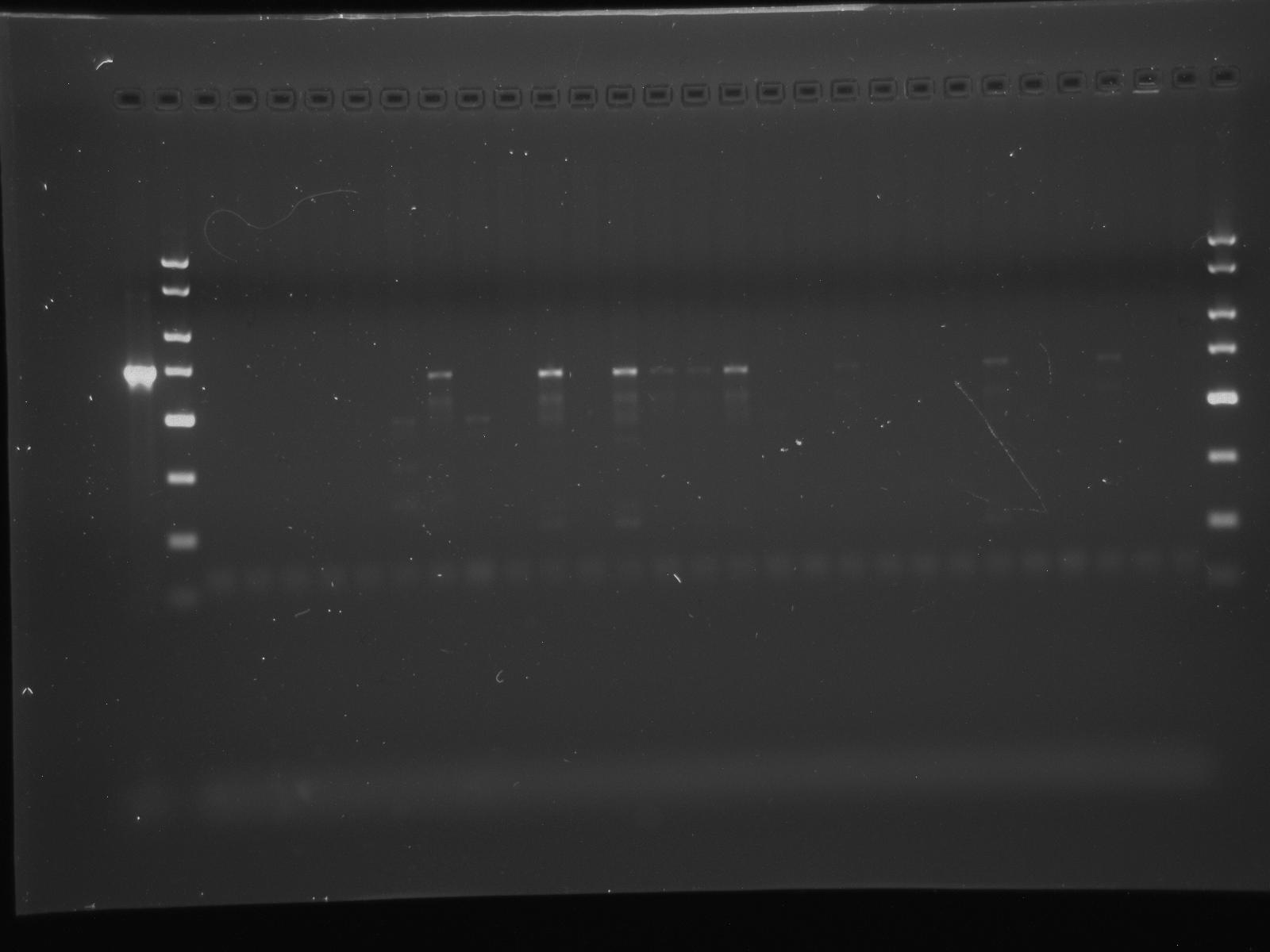


A.


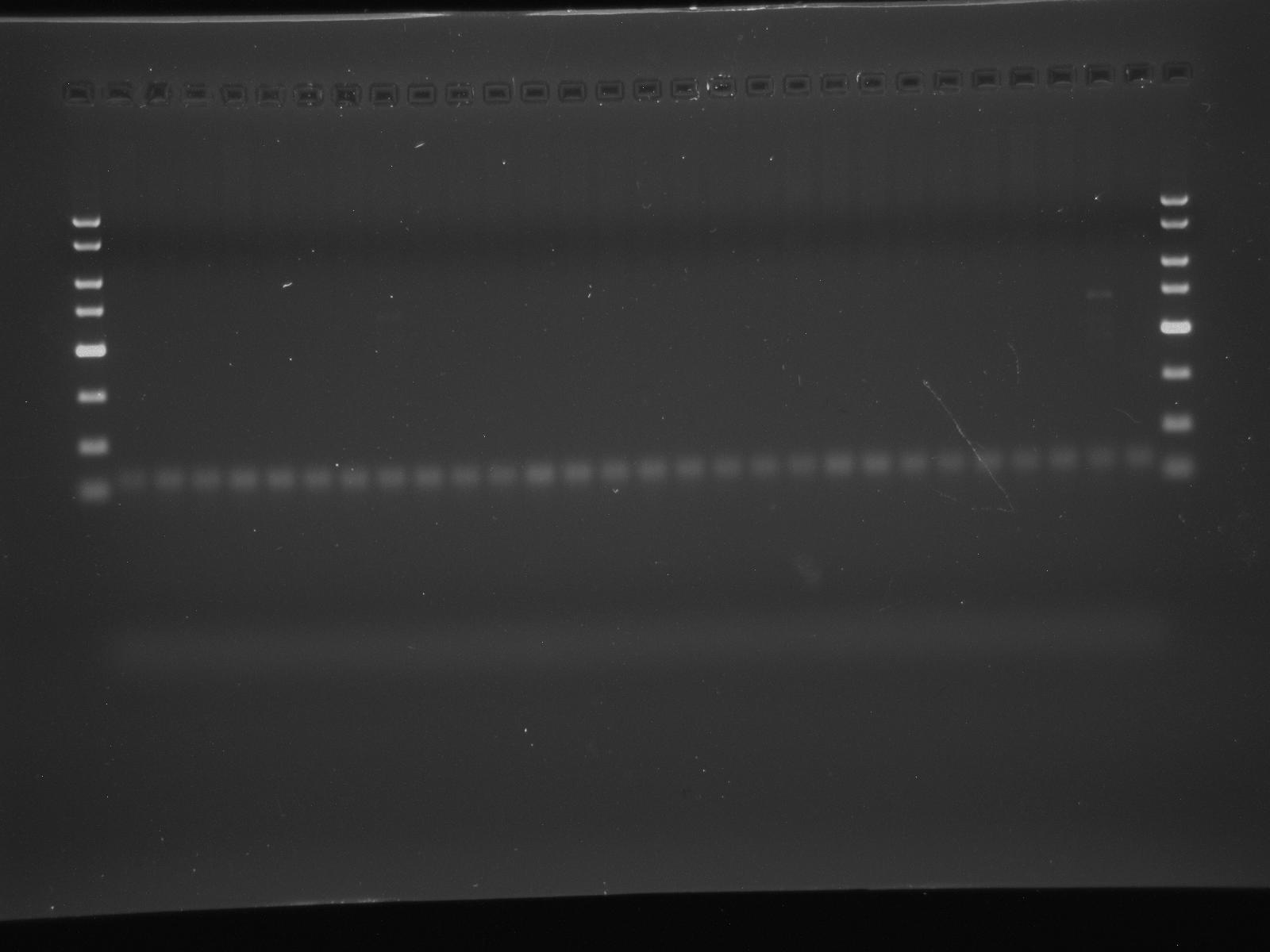


B.


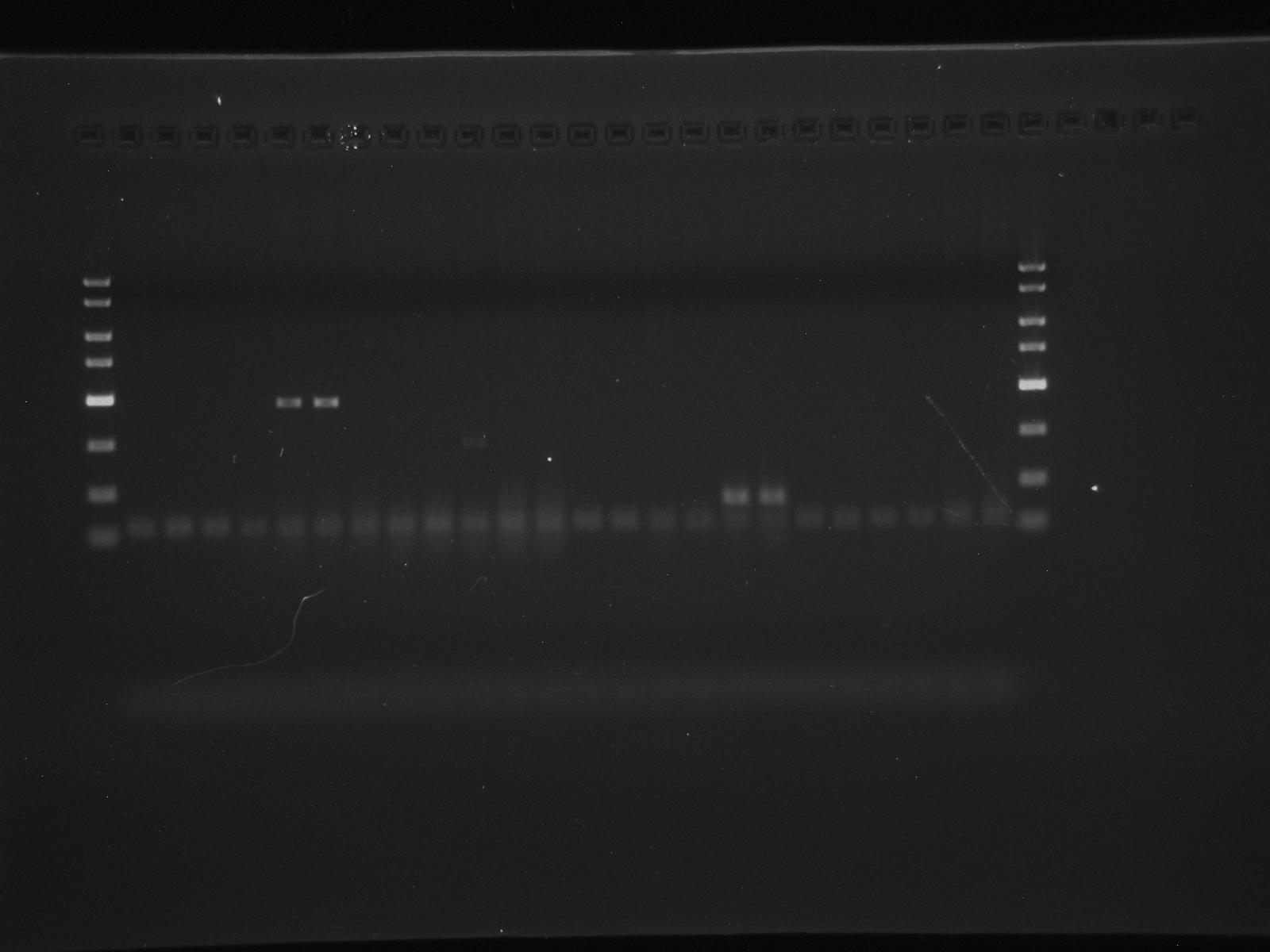


C.

Figure S3 (A, B, C). Agarose gel electrophoresis of amplified PCR products of the *cfr* gene (746 bp amplicon).


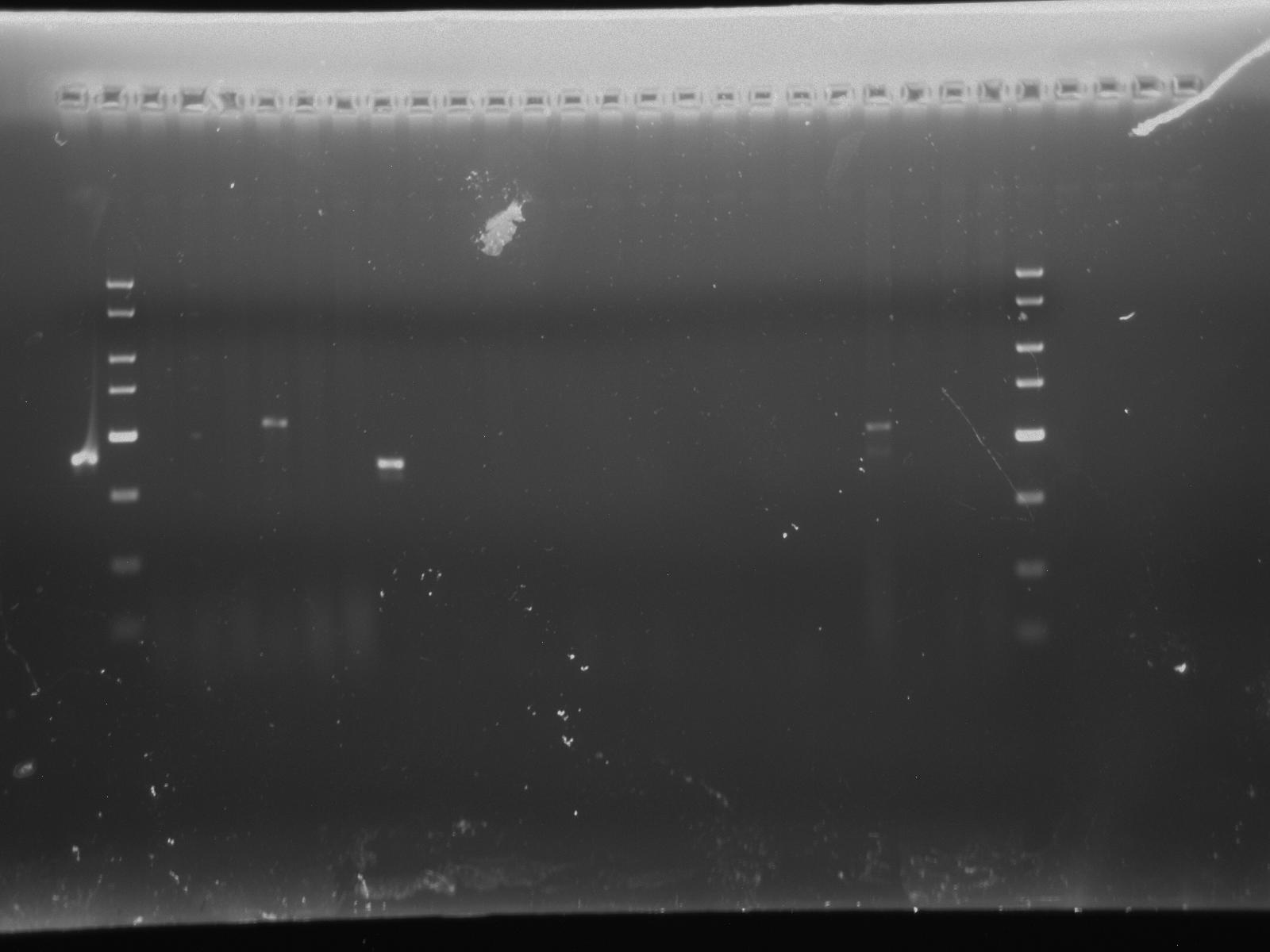


A.


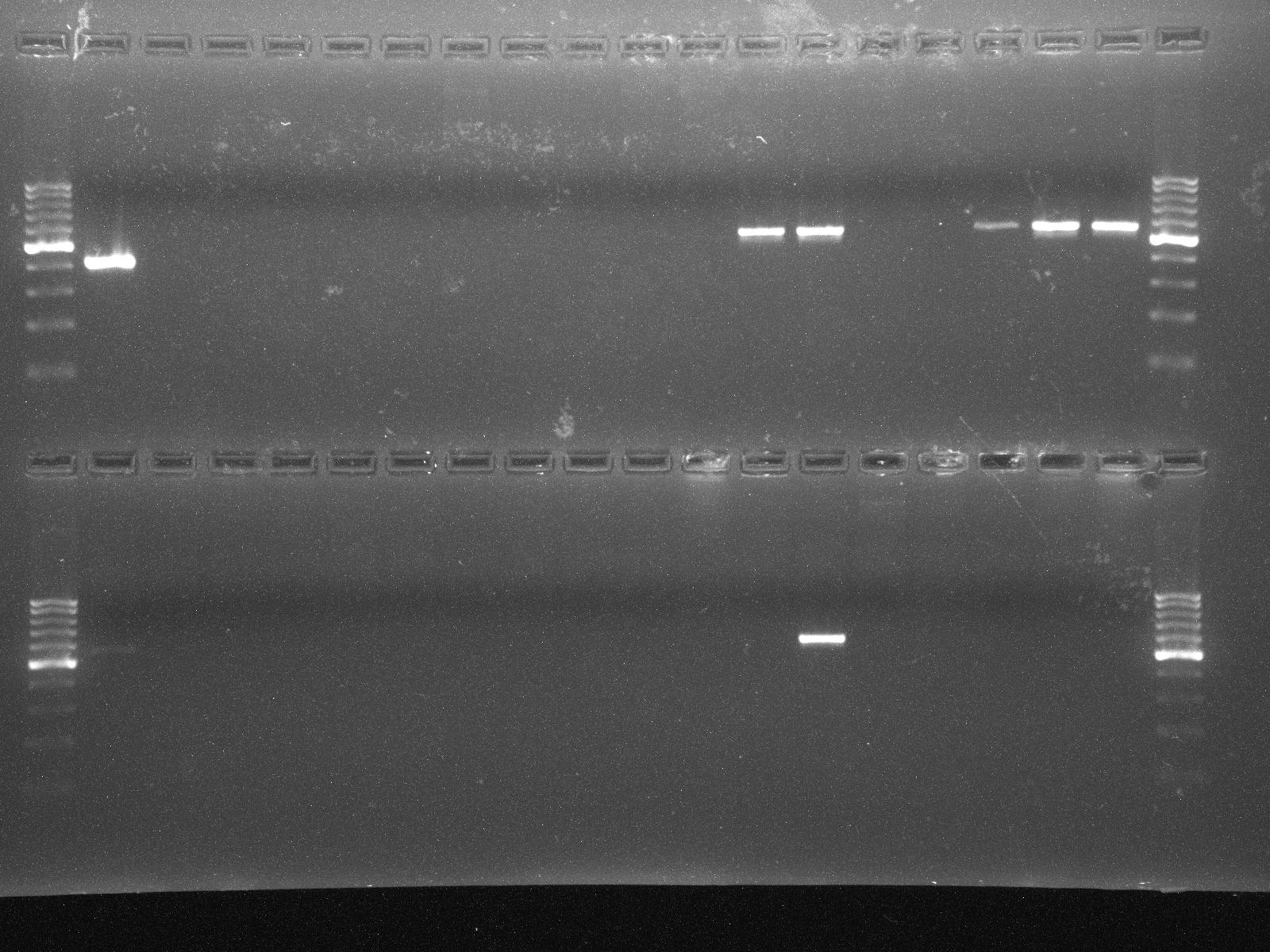


B.


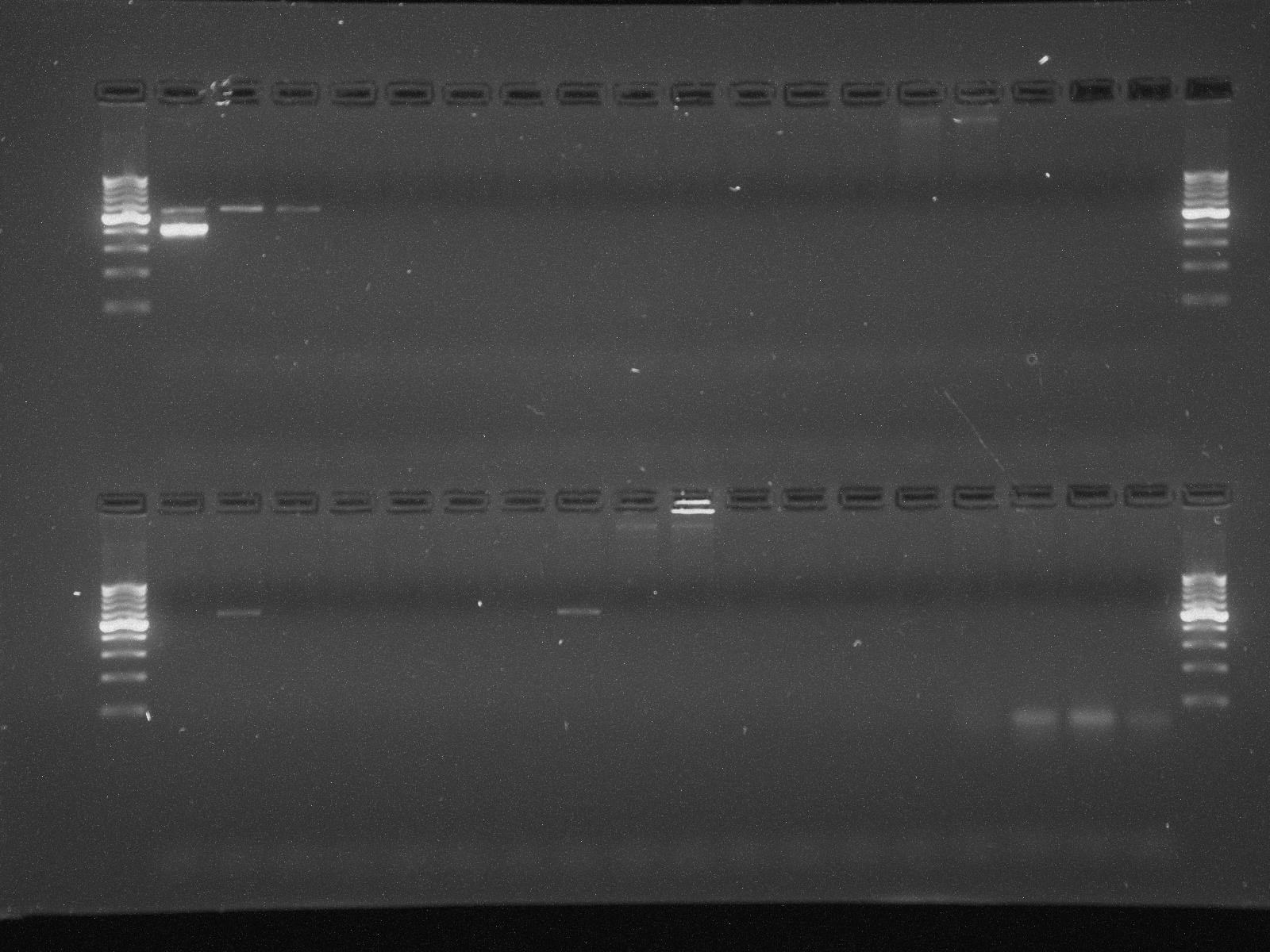


C.

Figure S4 (A, B, C). Duplex PCR amplification of the *ermA* (421 bp) and *ermC* (572 bp) genes.


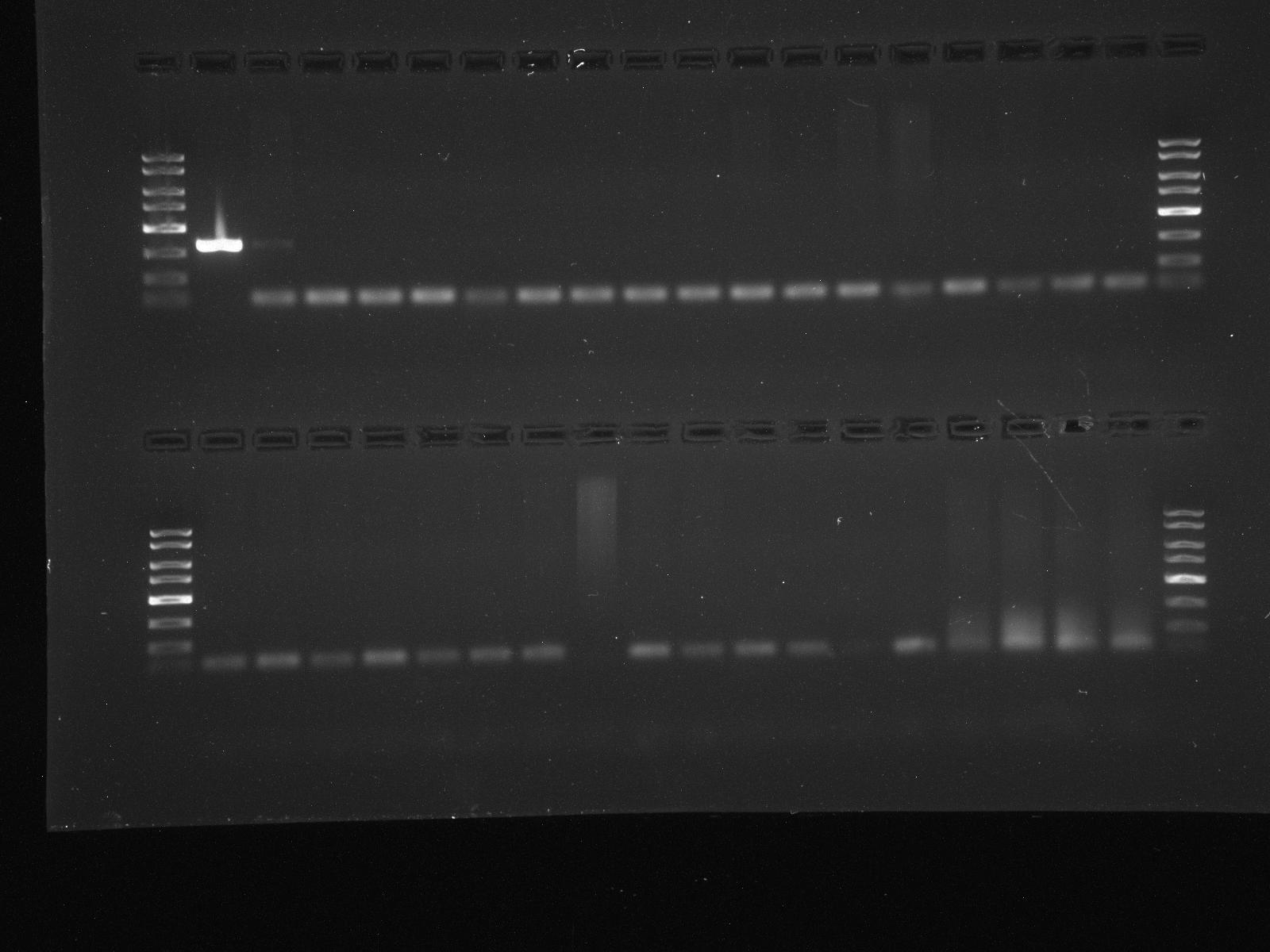


A.


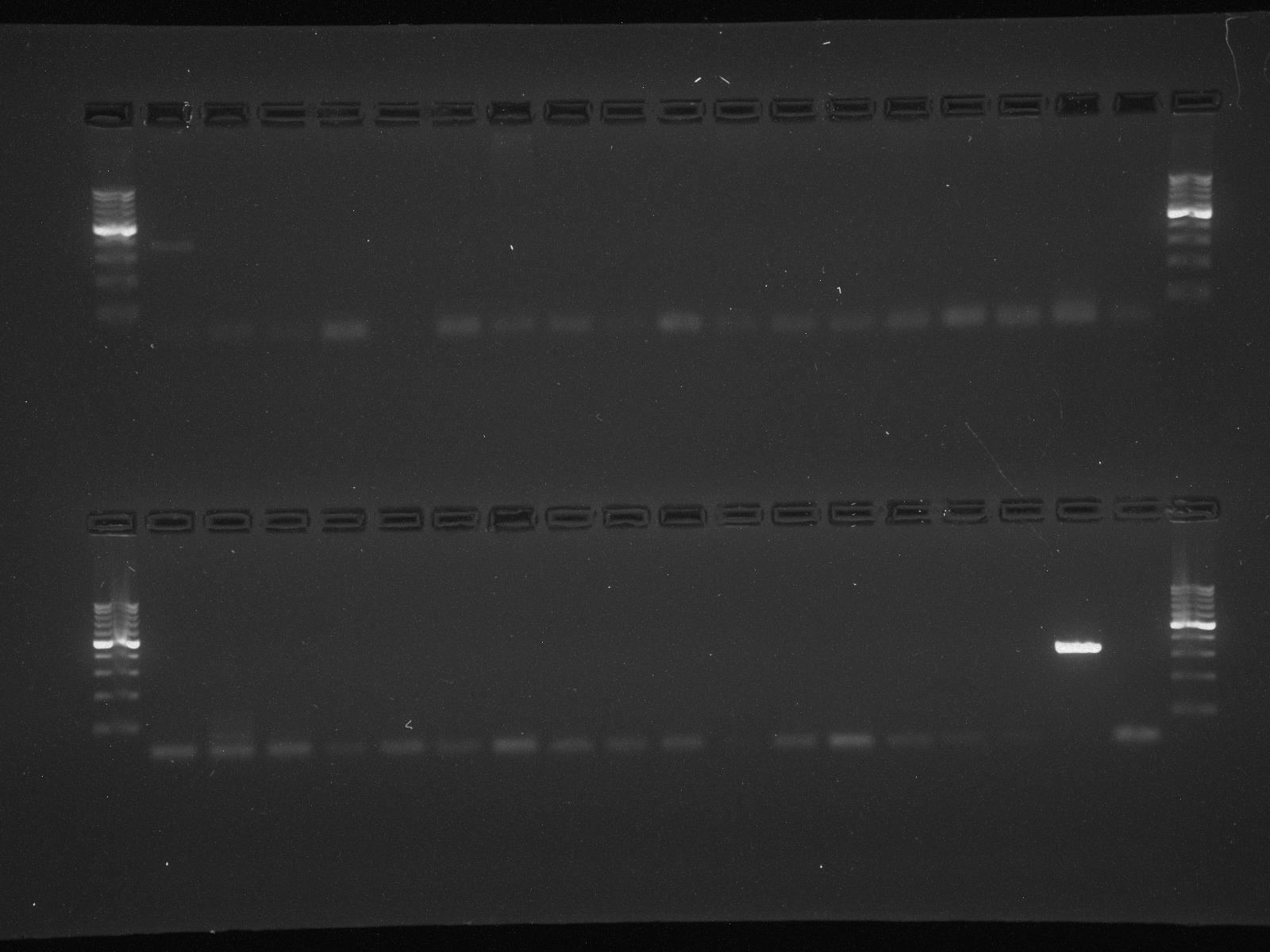


B.

Figure S5 (A, B). Agarose gel electrophoresis of amplified PCR products of the *ermB* gene (359 bp amplicon).


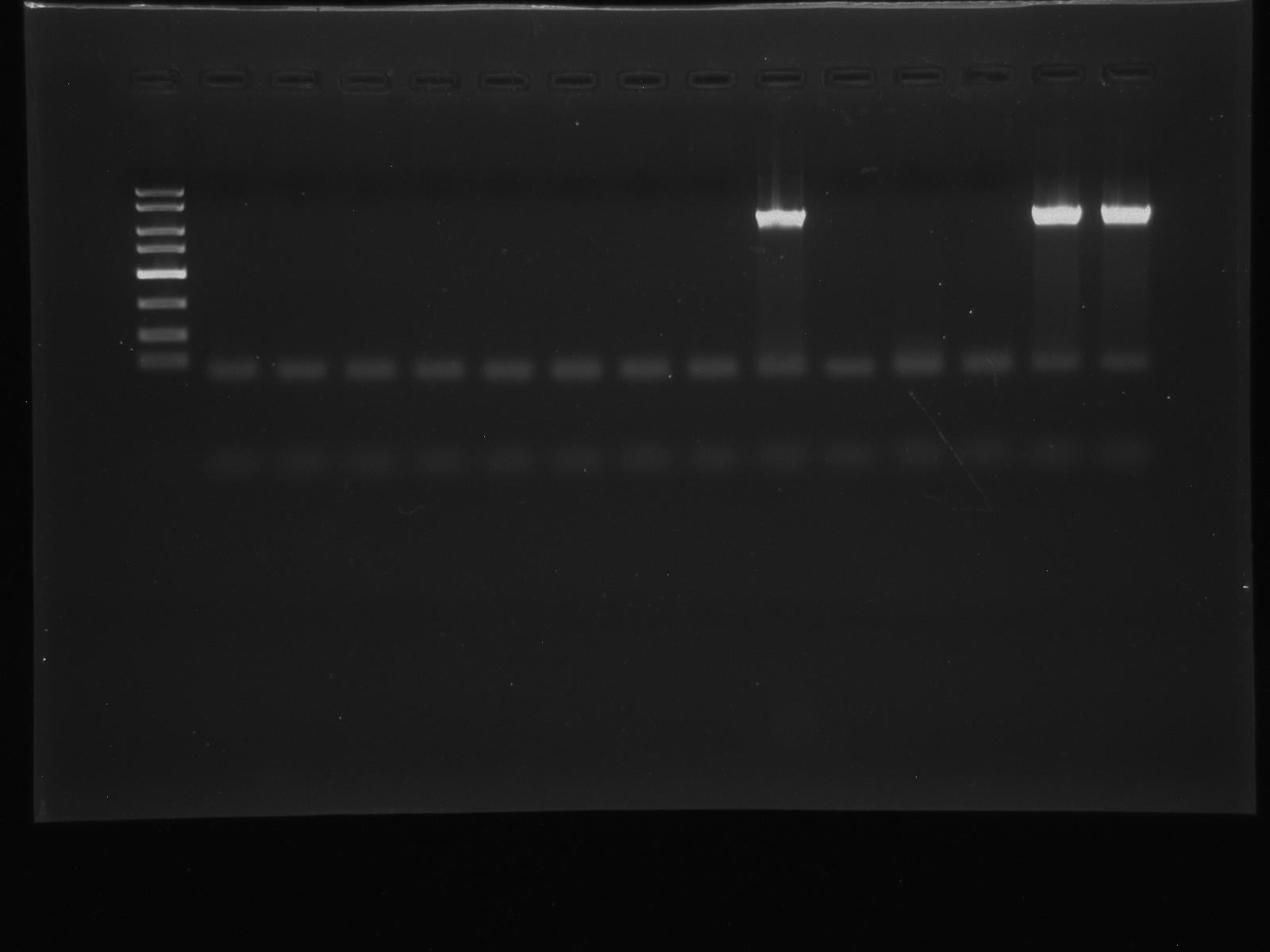


Figure S6. Agarose gel electrophoresis of amplified PCR products of the *fexA* gene (1272 bp amplicon).


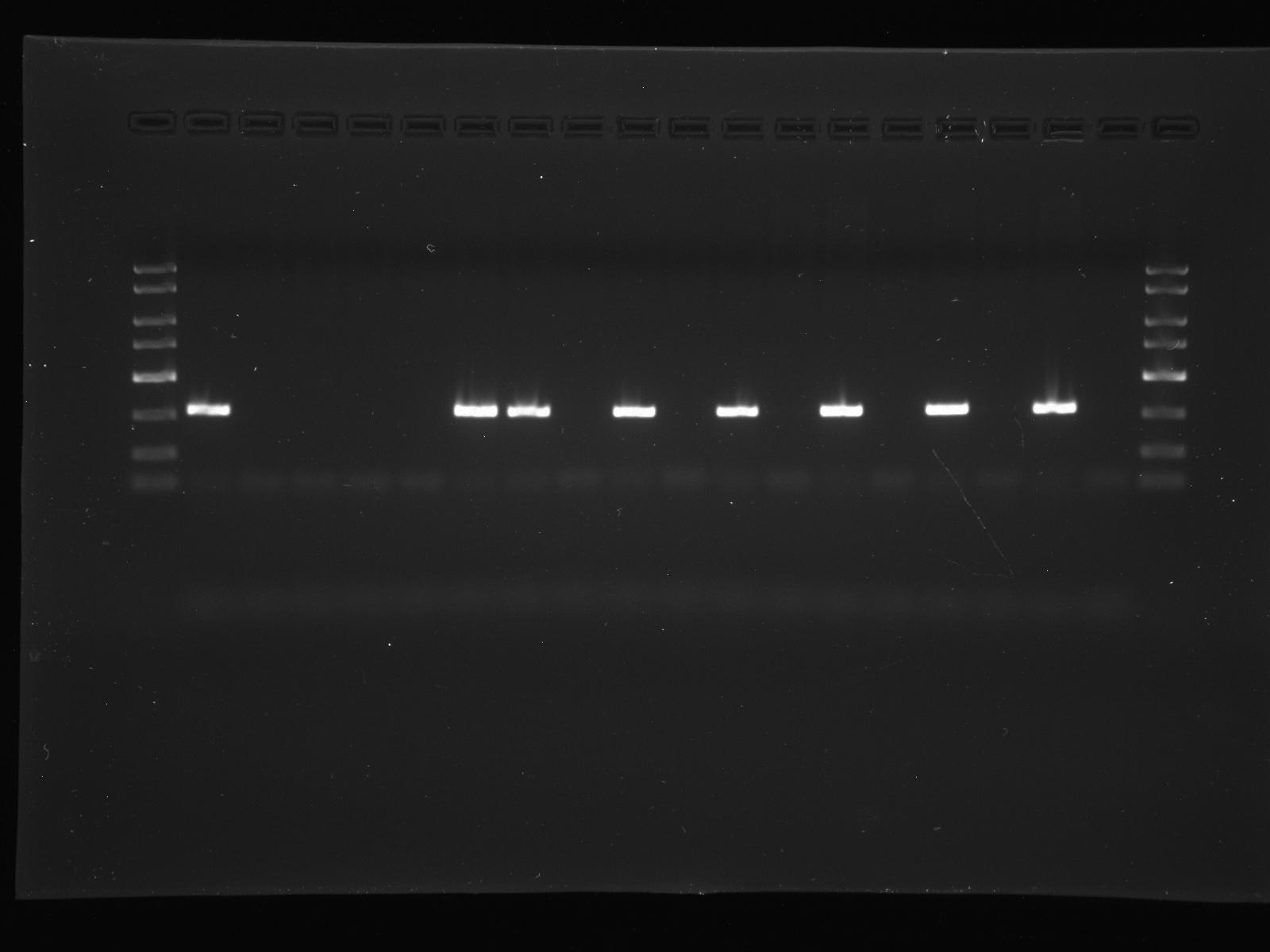


A.


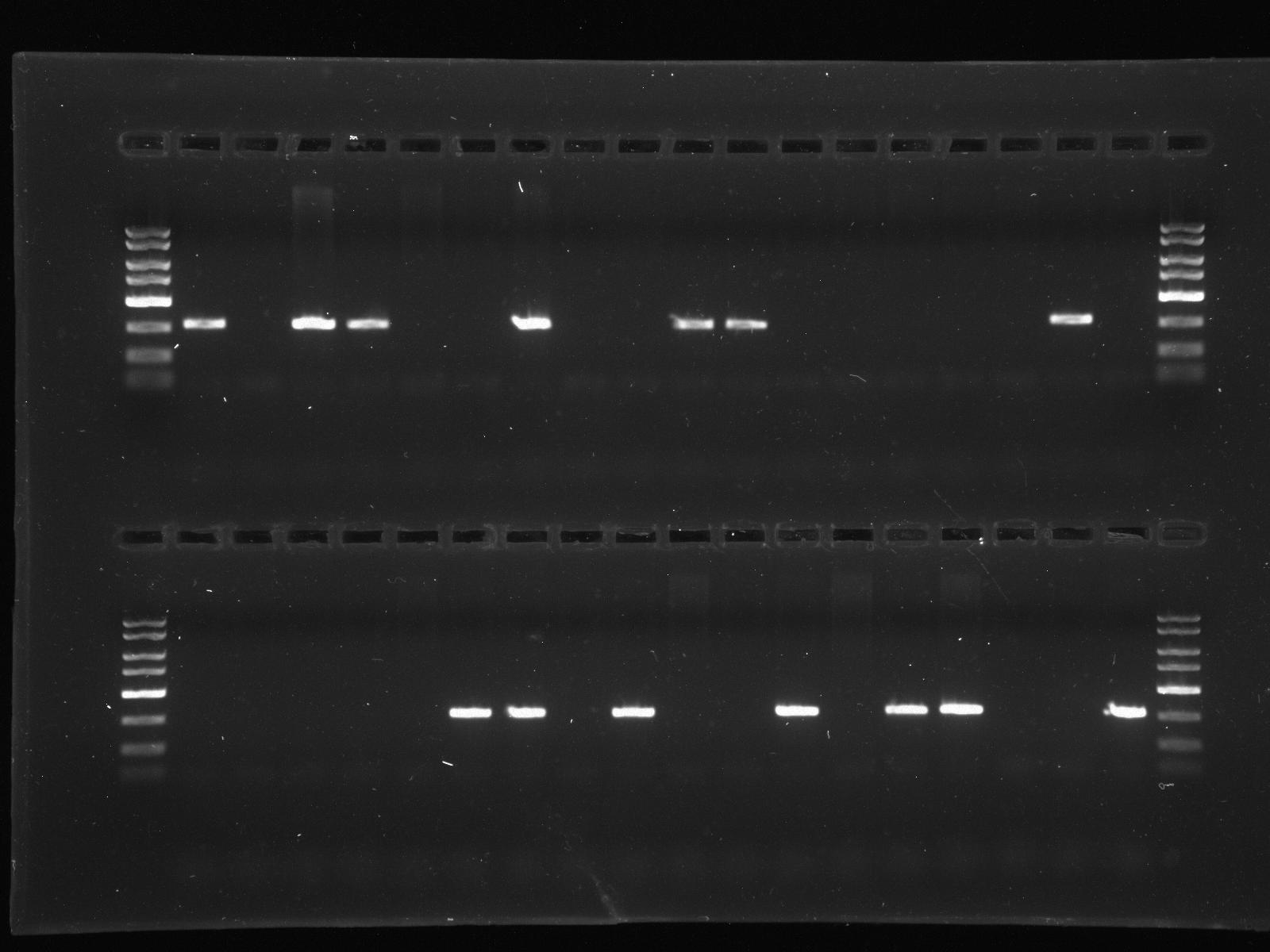


B.


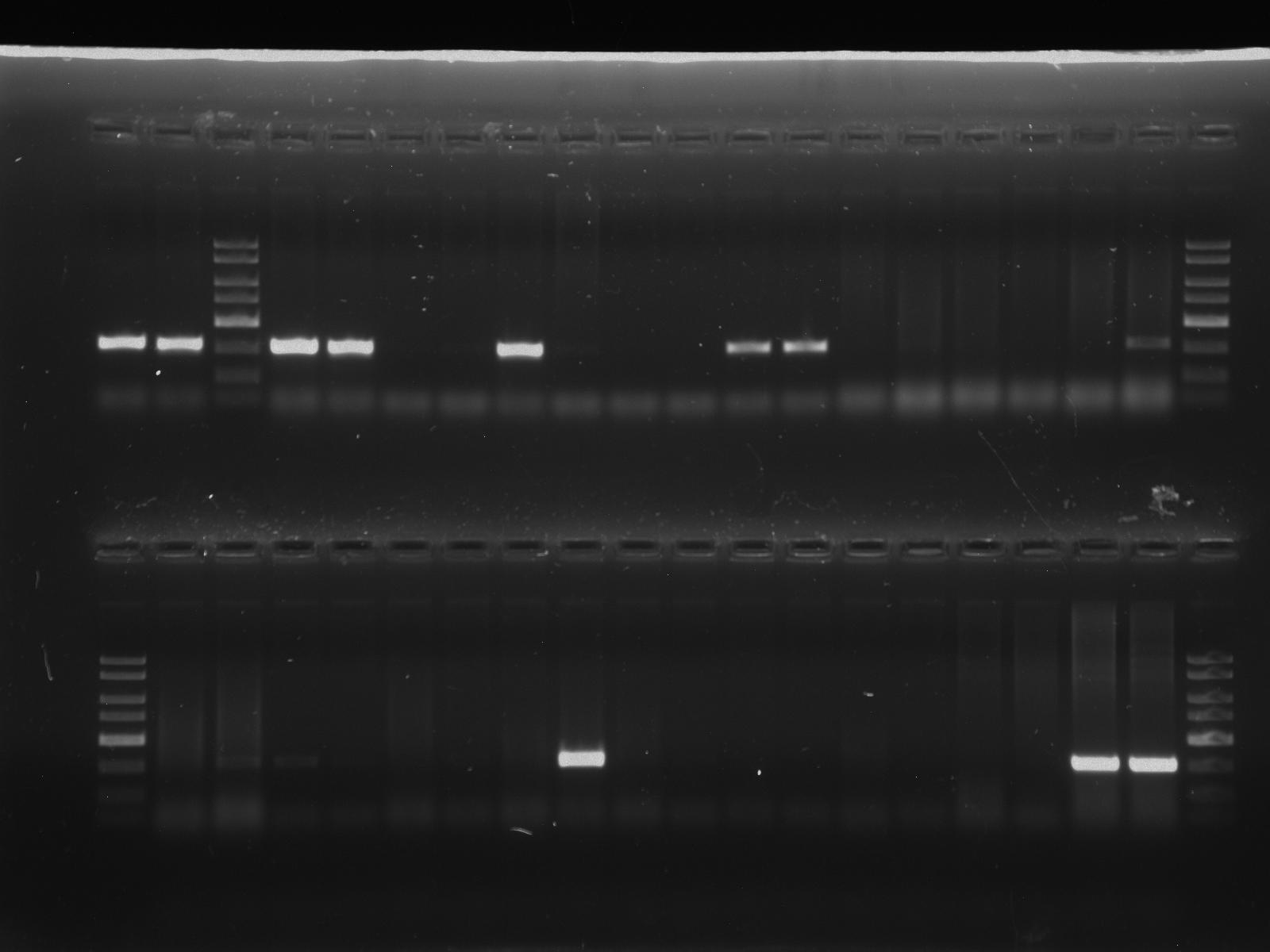


C.


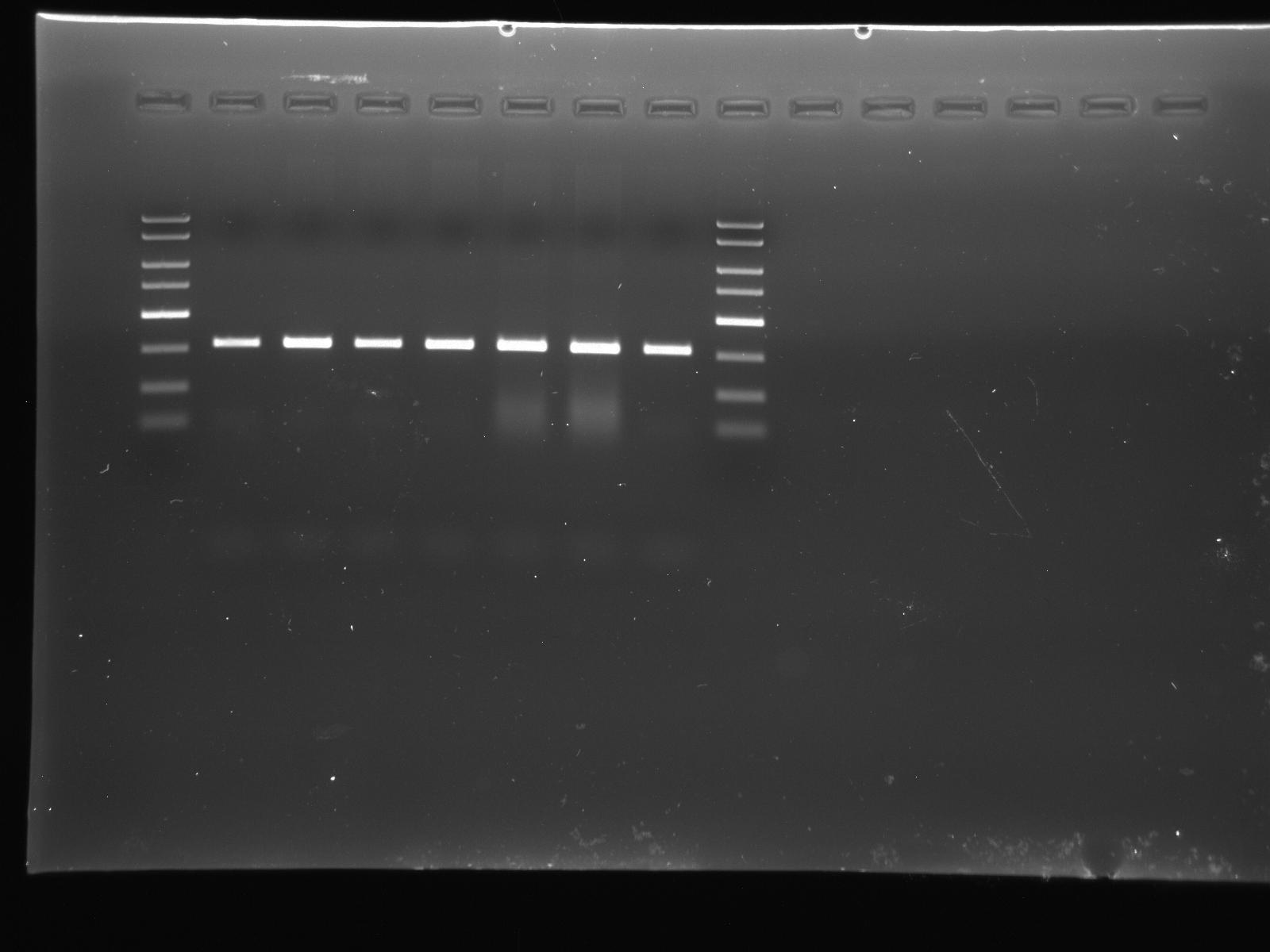


D.

Figure S7 (A, B, C, D). Agarose gel electrophoresis of amplified PCR products of the *lnuA* gene (323 bp amplicon).





A.


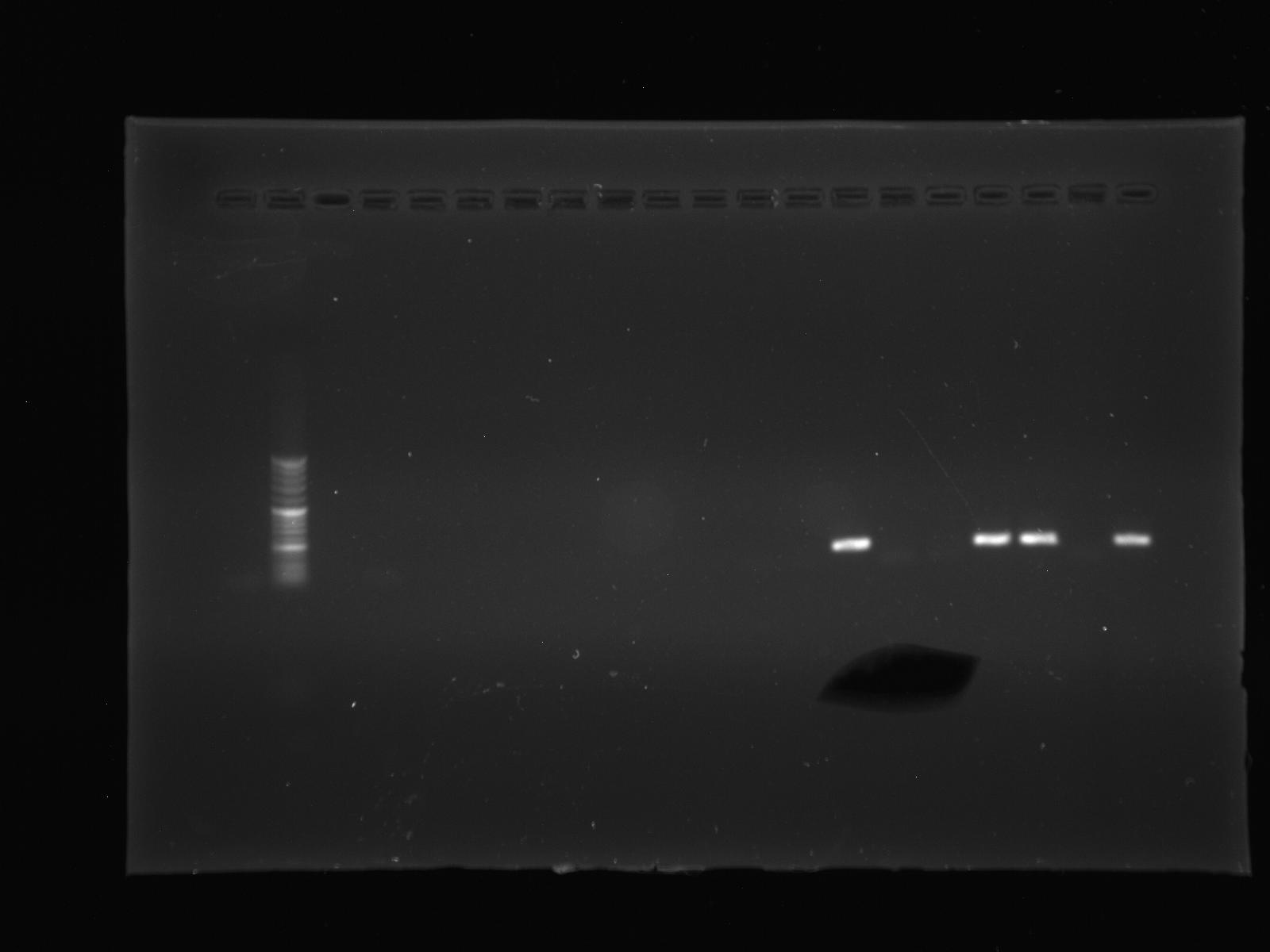


B.


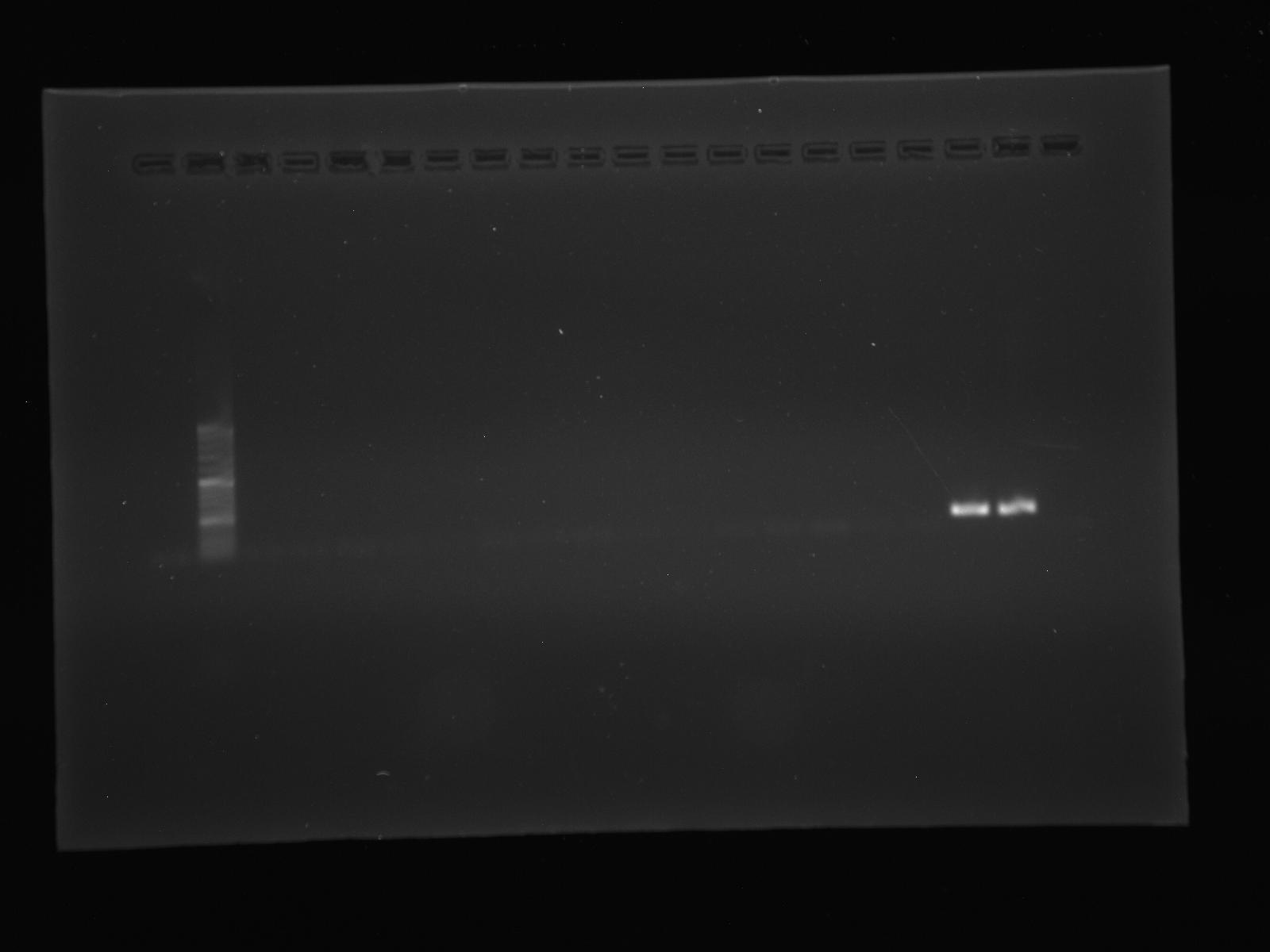


C.


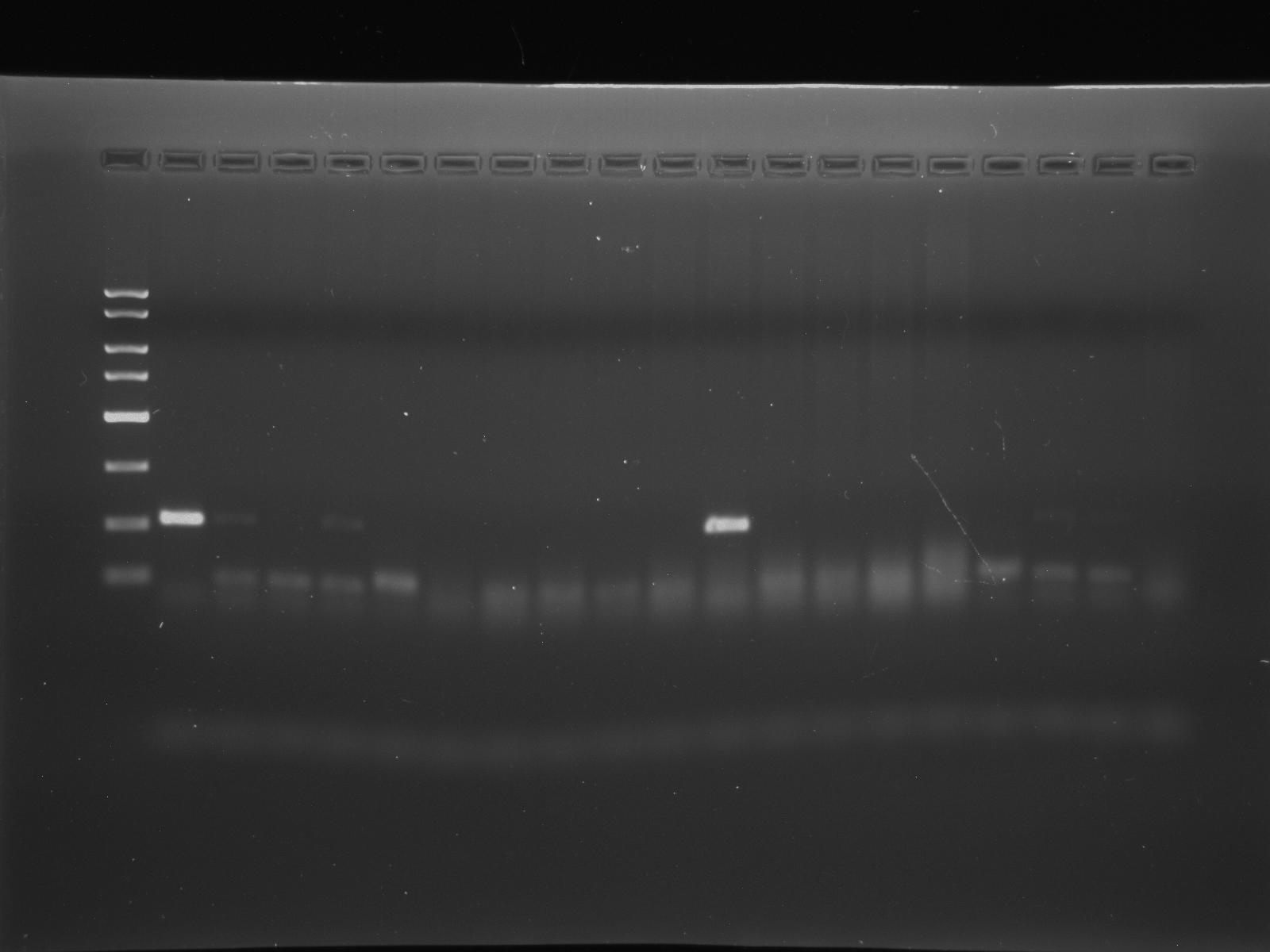


D.

Figure S8 (A, B, C, D). Agarose gel electrophoresis of amplified PCR products of the *mecA* gene ( bp amplicon).


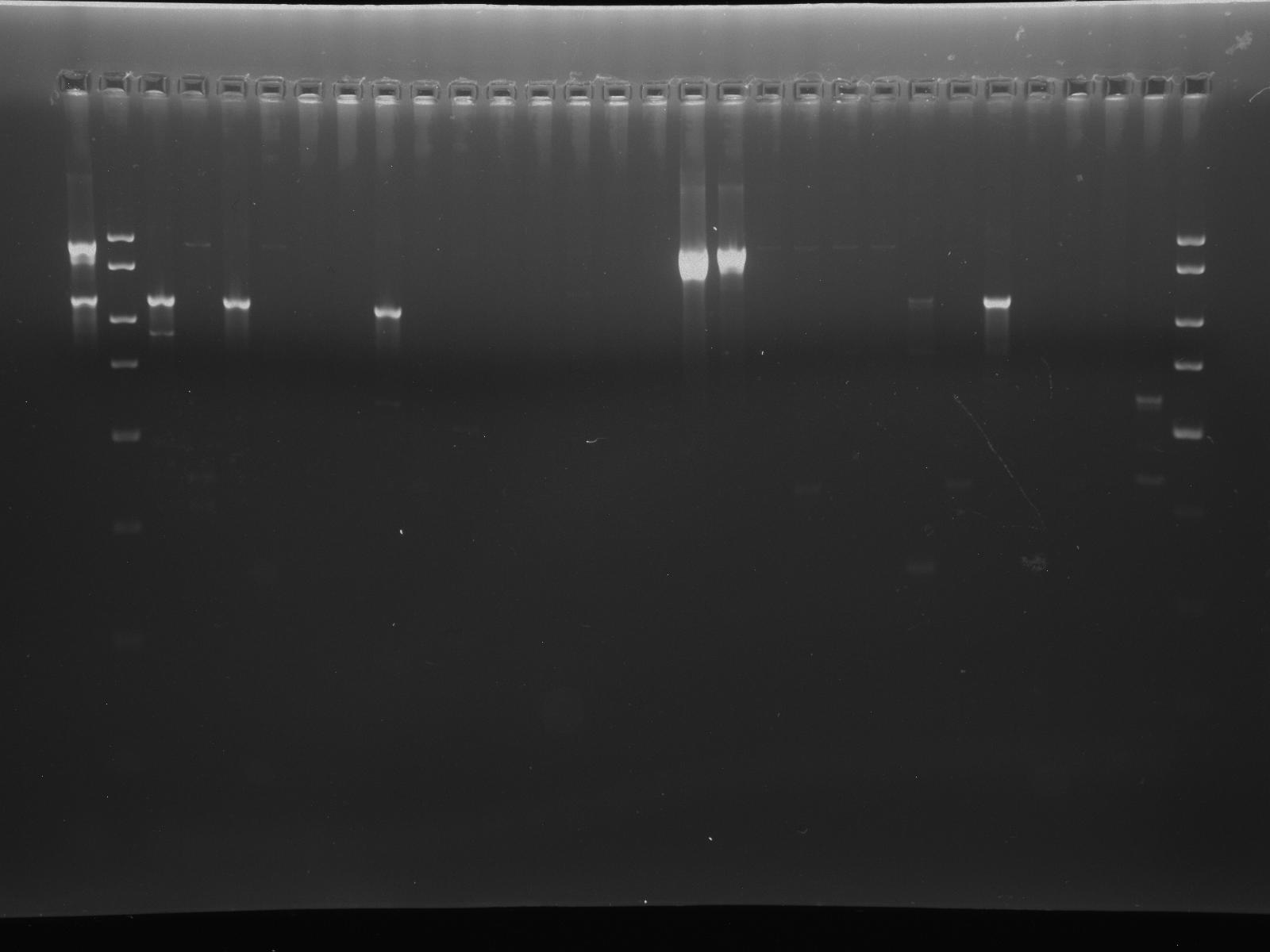


A.


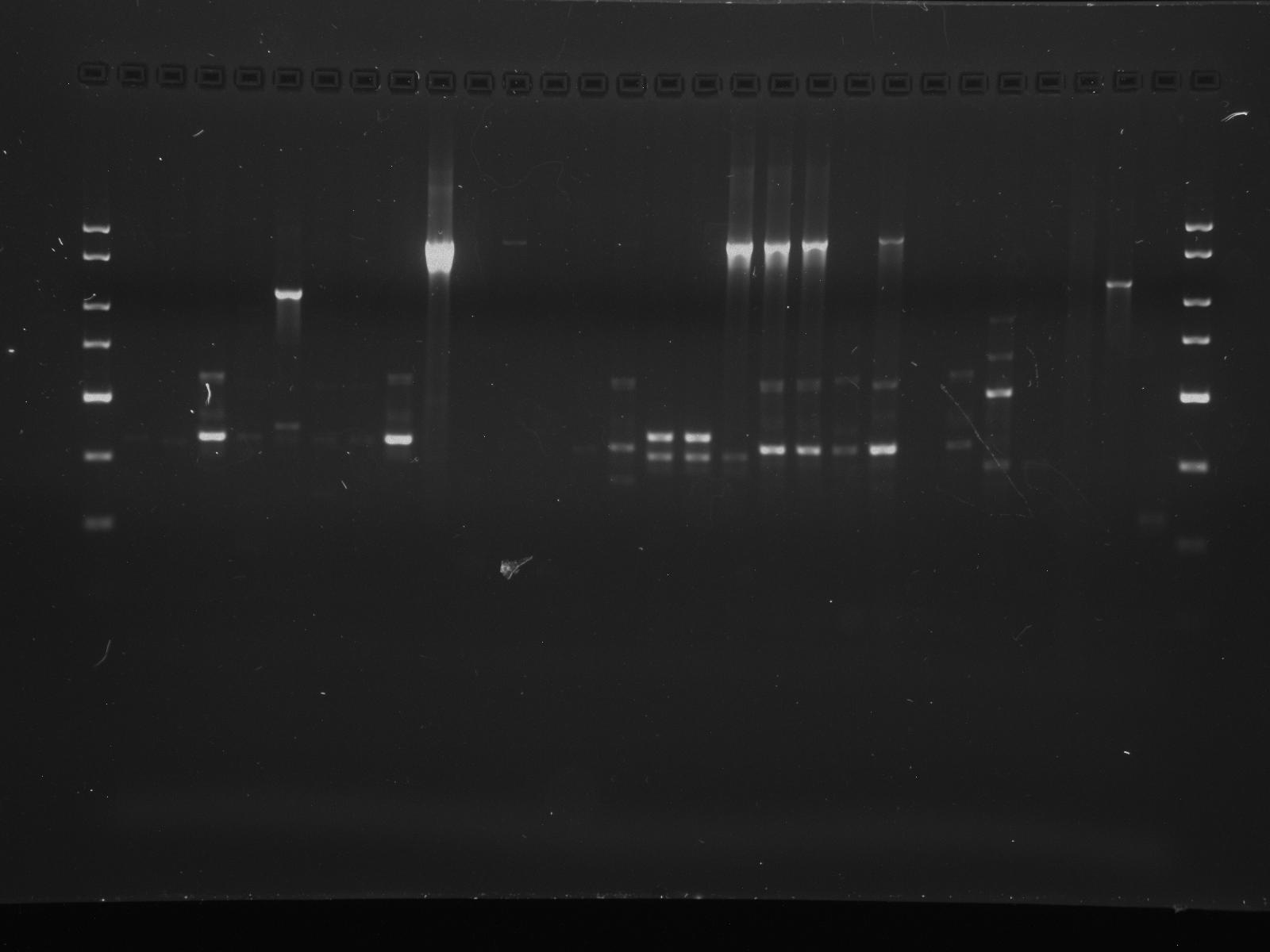


B.


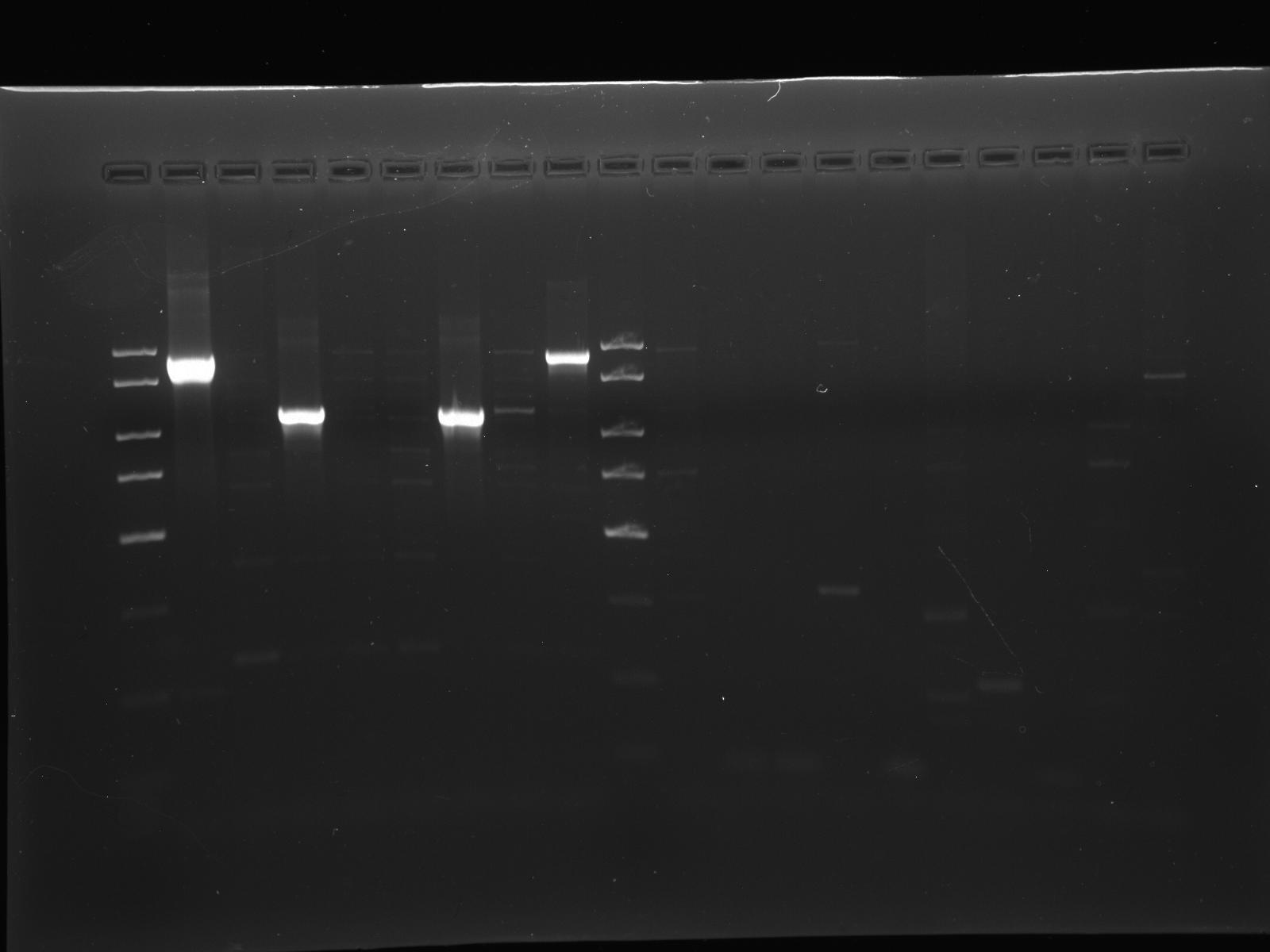


C.


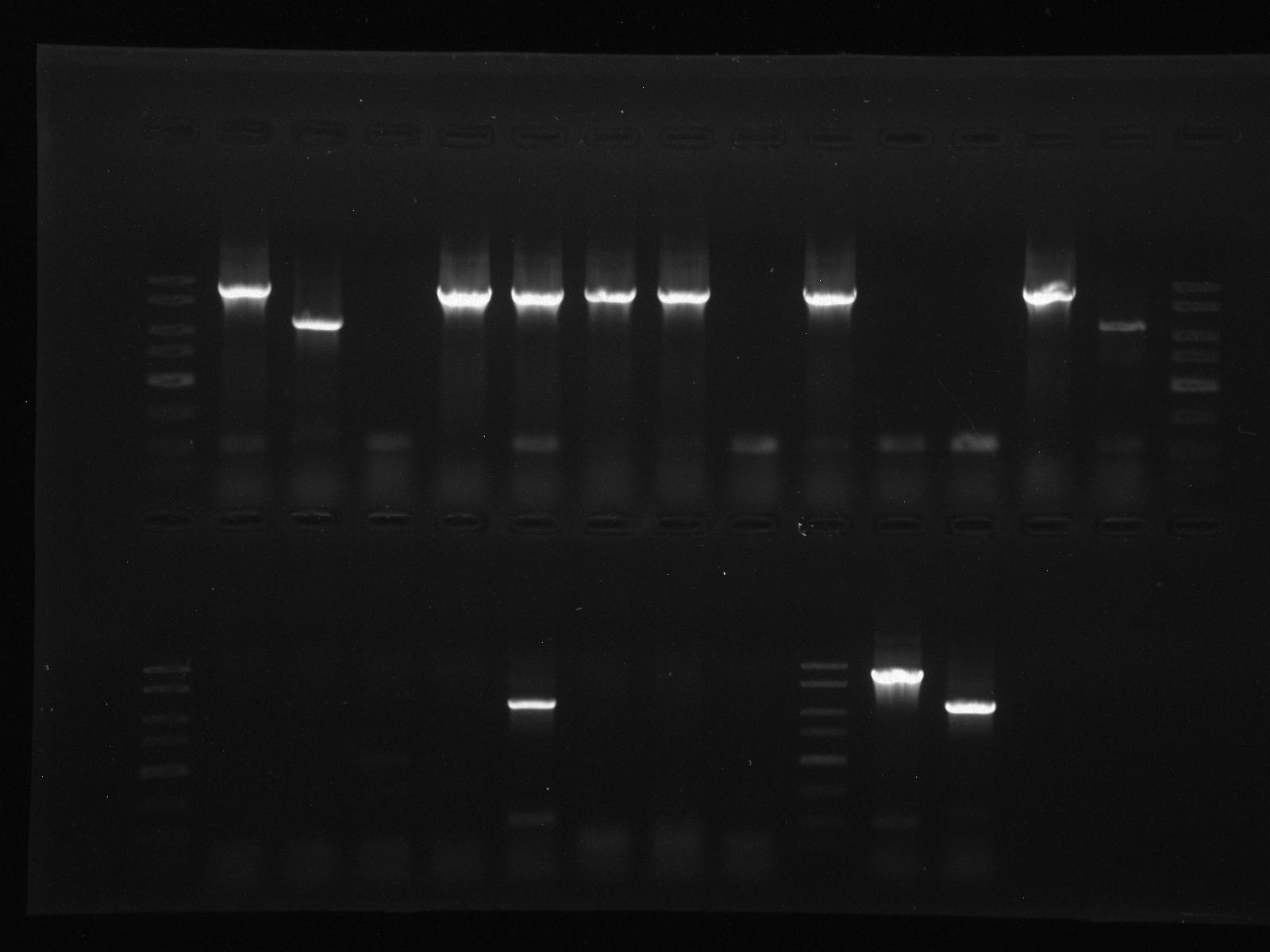


D.

Figure S9 (A, B, C, D). Duplex PCR amplification of the *tetK* (1159 bp) and *tetM* (1862 bp) genes.


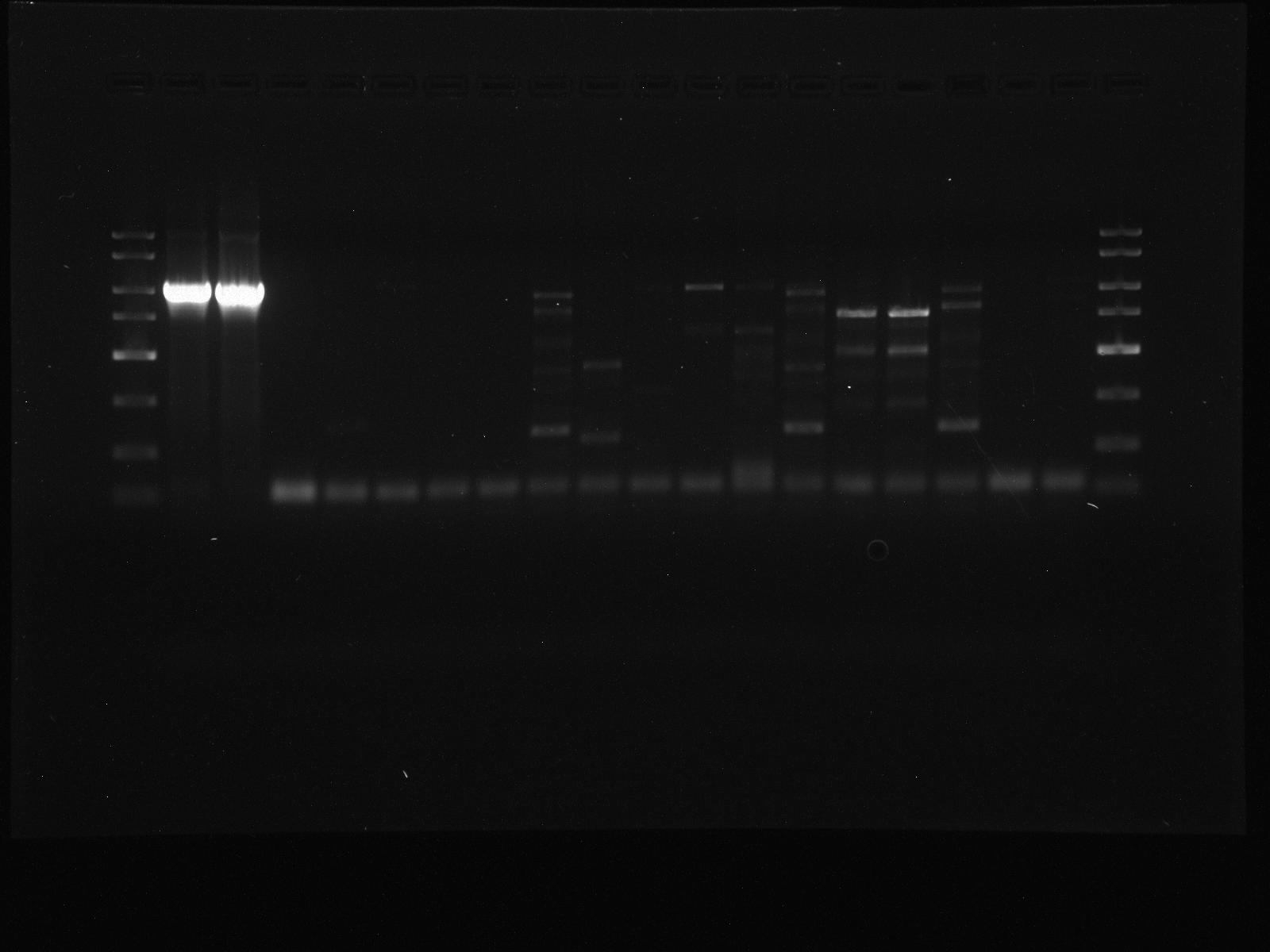


A.


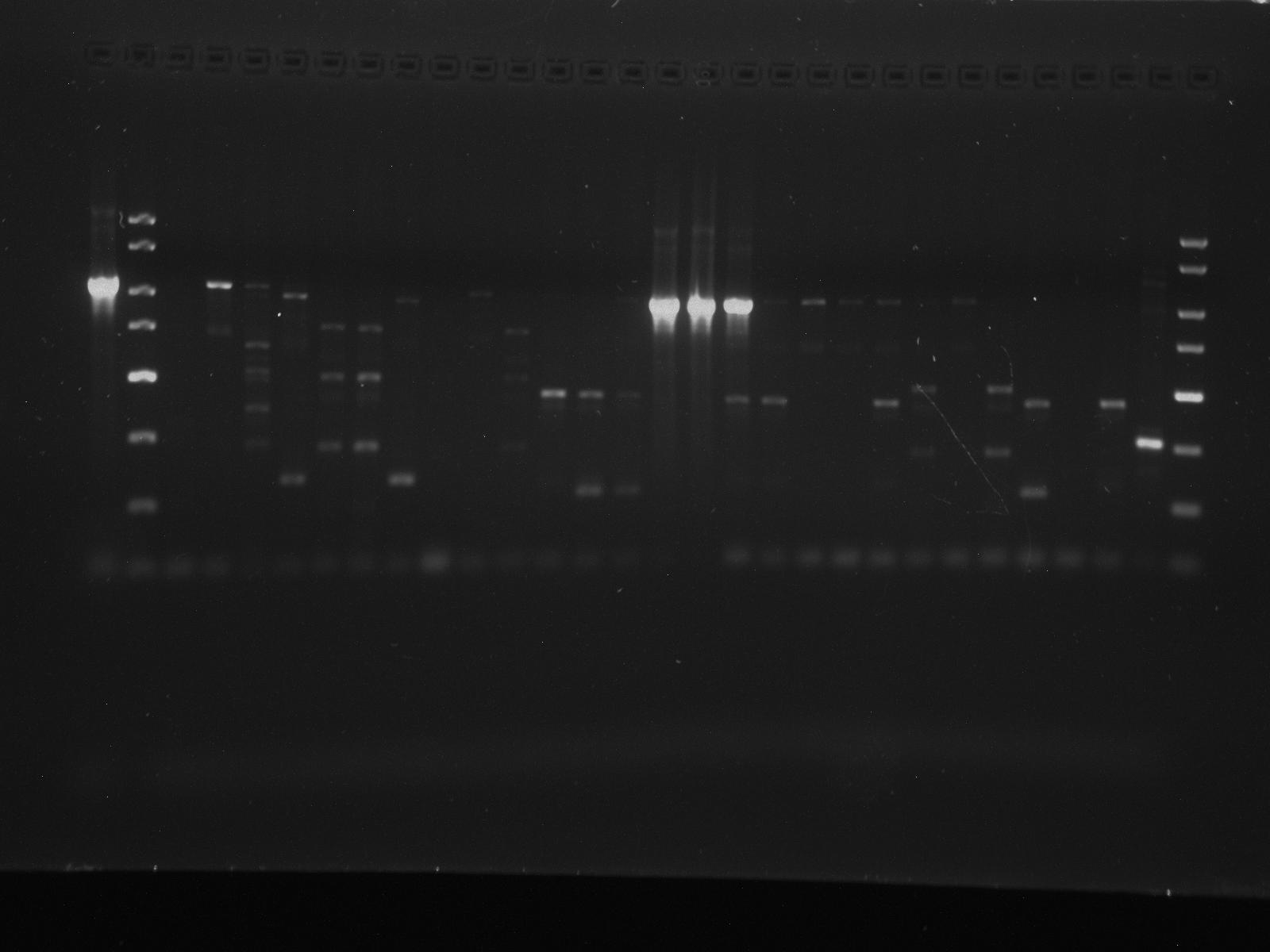


B.


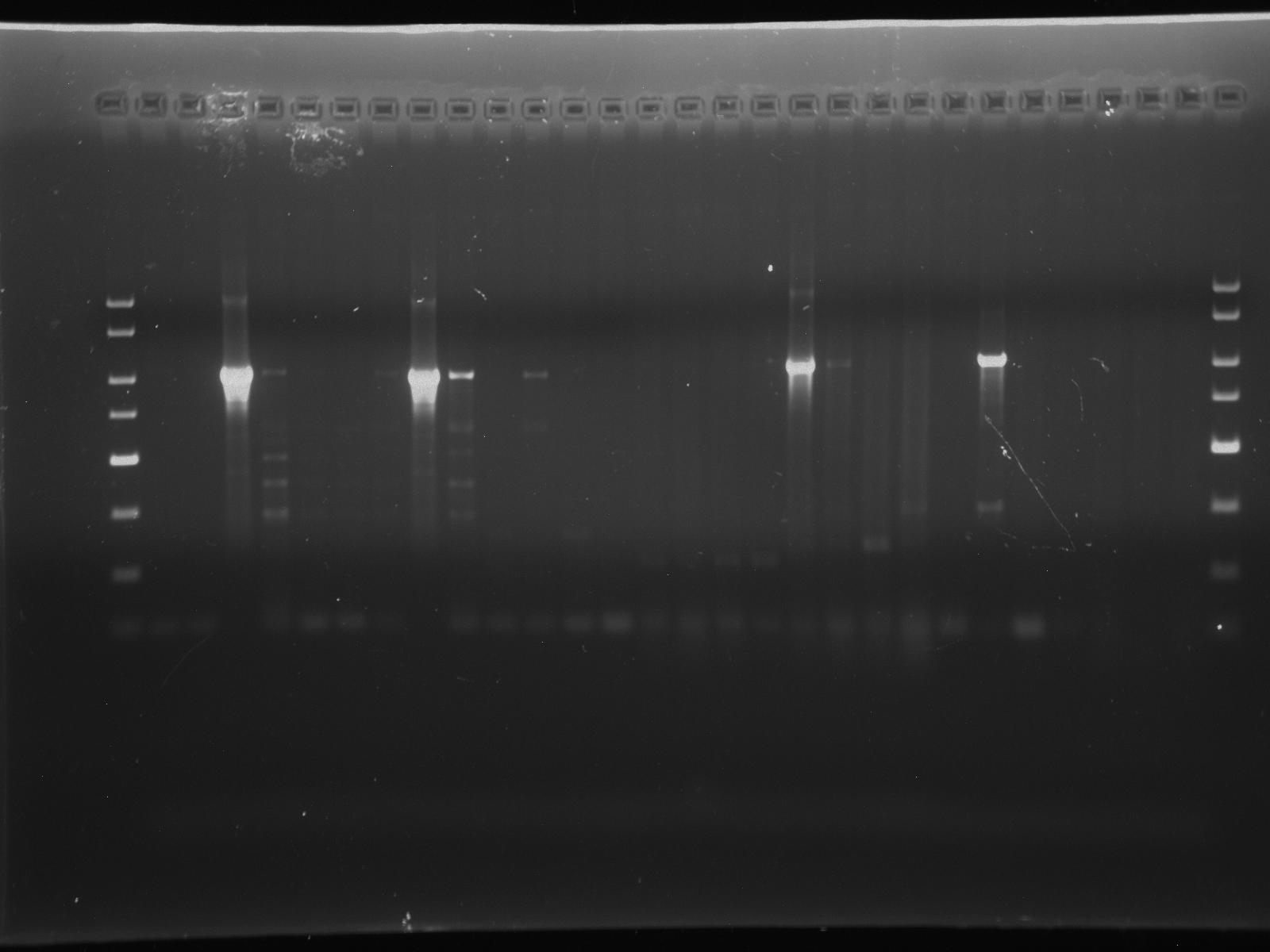


C.


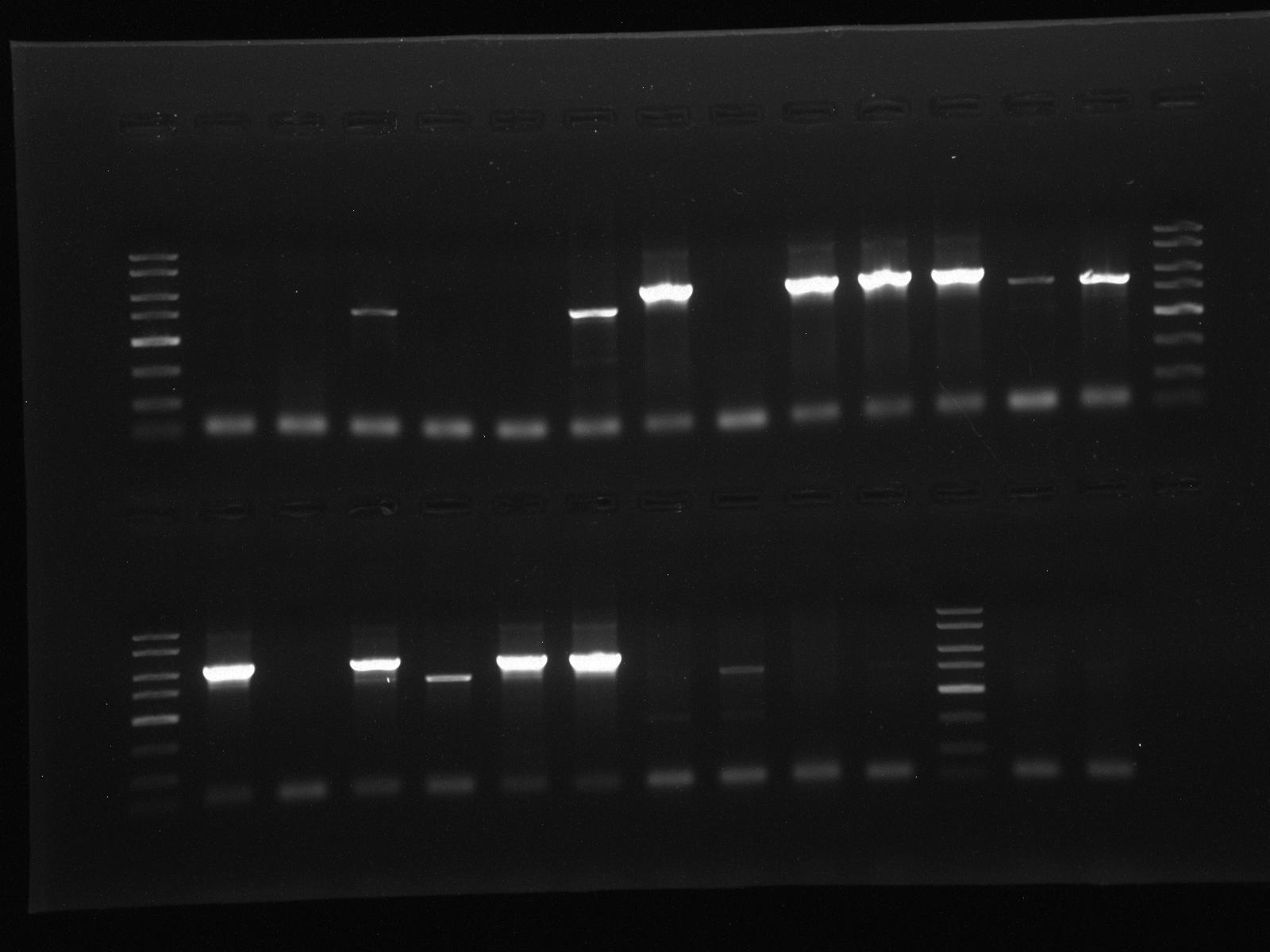


D.

Figure S10 (A, B, C, D). Agarose gel electrophoresis of amplified PCR products of the *tetL* gene (1077 bp amplicon).


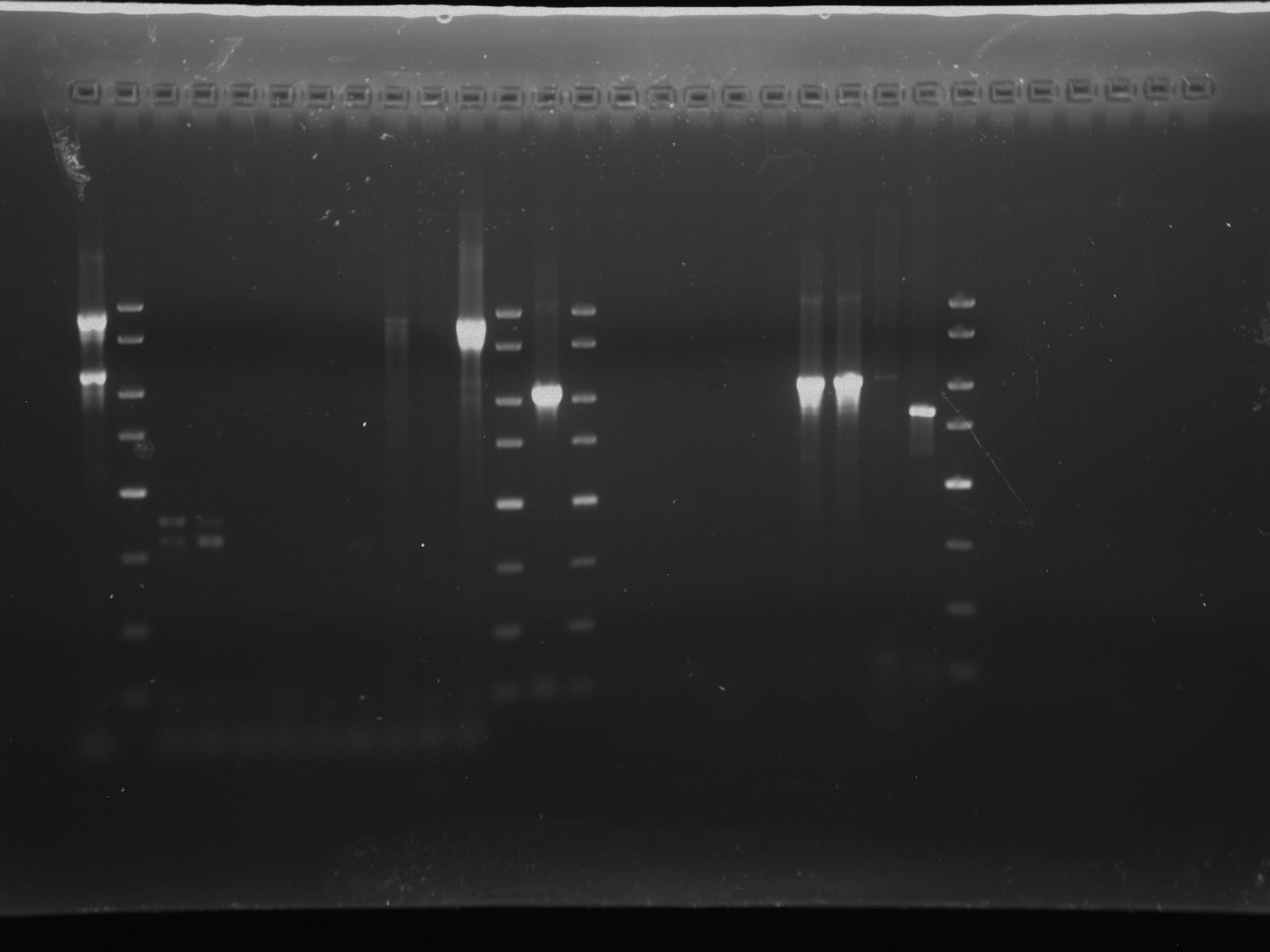


Figure S11. Duplex PCR amplification of the *tetK* (1159 bp) and *tetM* (1862 bp) genes and amplified PCR products of the *tetL* gene (1077 bp).


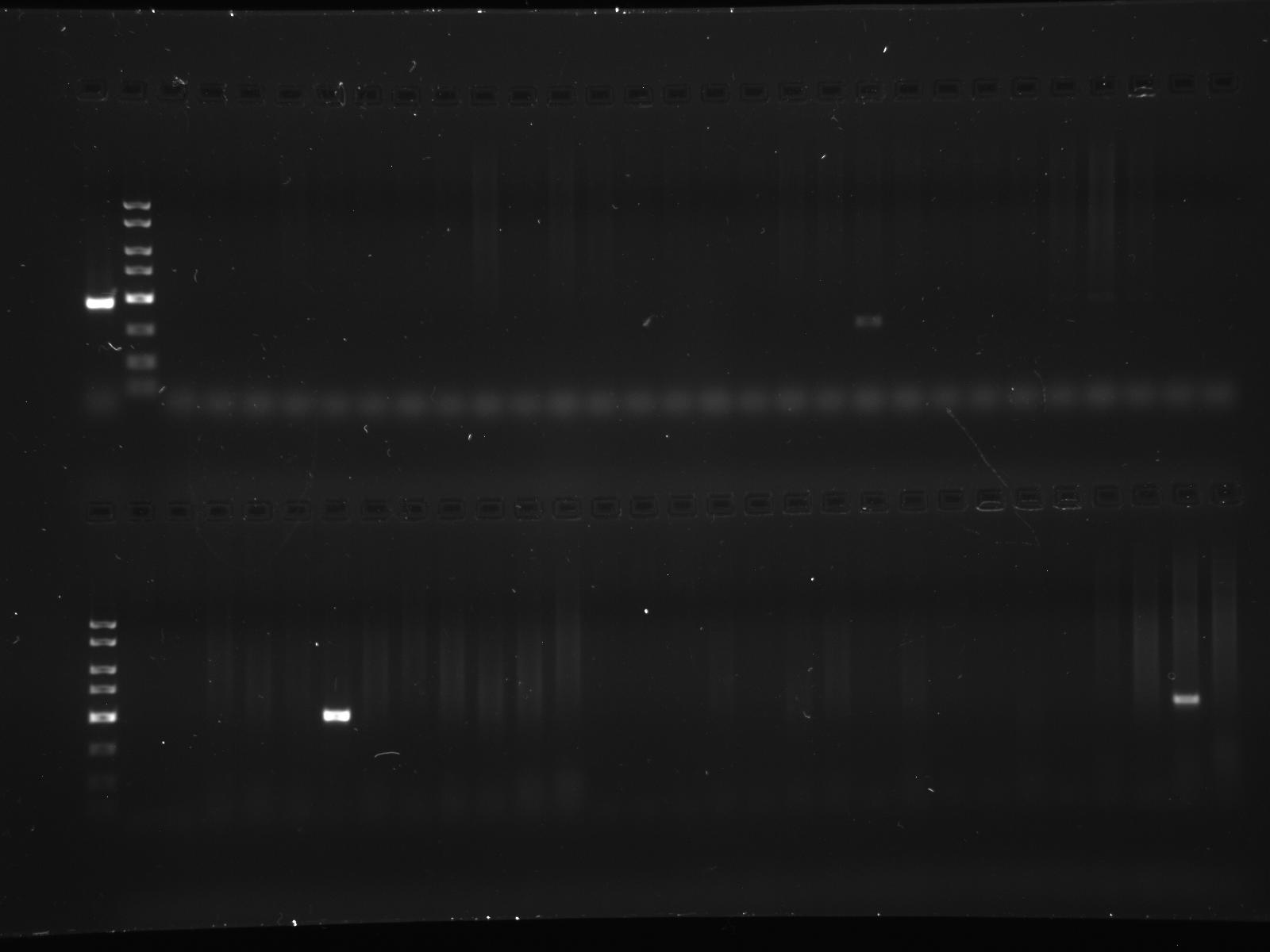


A.


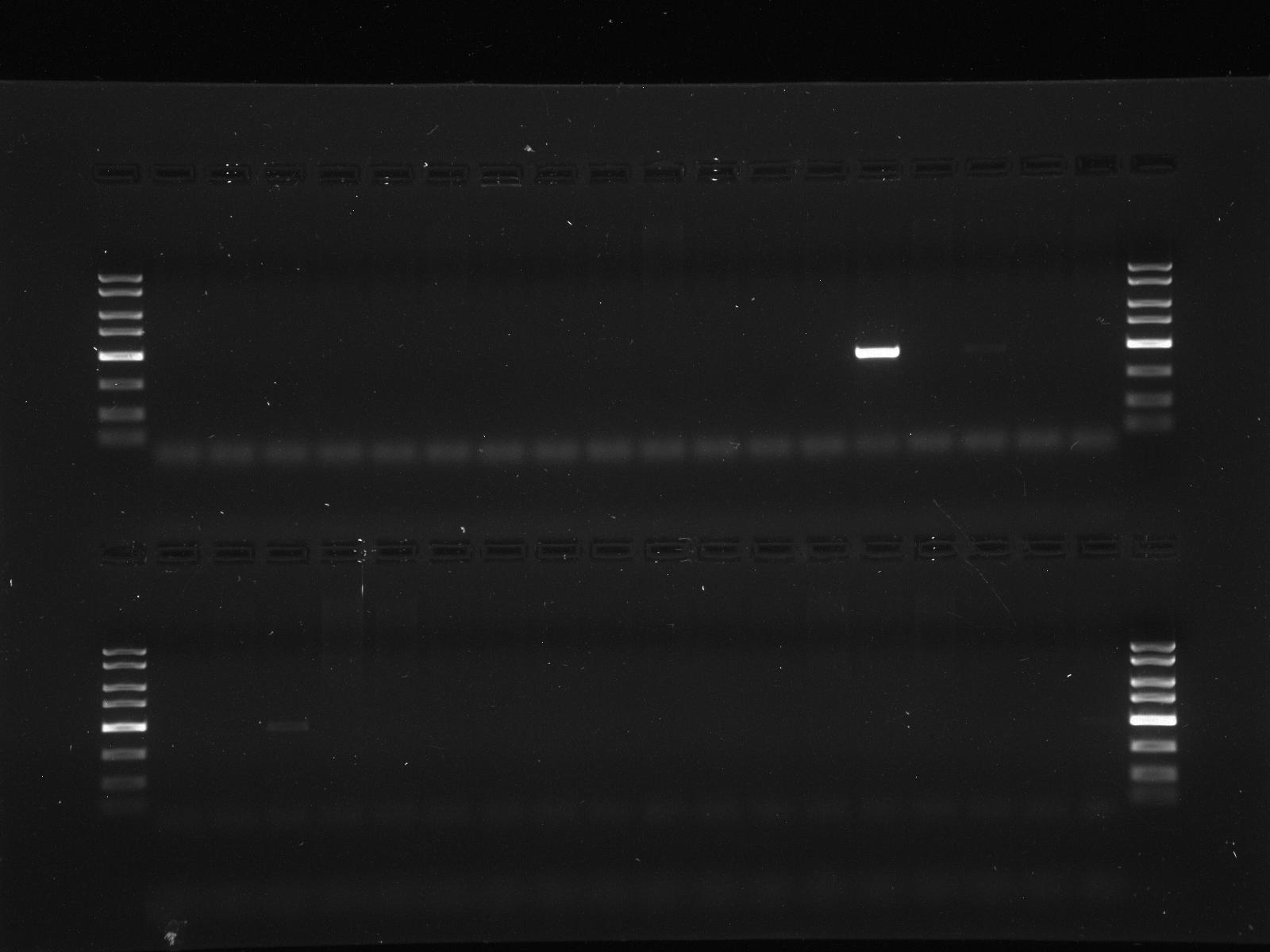


B.

Figure S12. Agarose gel electrophoresis of amplified PCR products of the *vga* gene (470 bp amplicon).
